# Novel Biomarkers of Atherosclerotic Vascular Disease—Latest Insights in the Research Field

**DOI:** 10.3390/ijms23094998

**Published:** 2022-04-30

**Authors:** Cristina Andreea Adam, Delia Lidia Șalaru, Cristina Prisacariu, Dragoș Traian Marius Marcu, Radu Andy Sascău, Cristian Stătescu

**Affiliations:** 1 Institute of Cardiovascular Diseases “Prof. Dr. George I.M. Georgescu”, 700503 Iași, Romania; adam.cristina93@gmail.com (C.A.A.); prisacariu88@yahoo.com (C.P.); radu.sascau@gmail.com (R.A.S.); cstatescu@gmail.com (C.S.); 2 Department of Internal Medicine, University of Medicine and Pharmacy “Grigore T. Popa”, 700115 Iași, Romania; dragos.marcu11@yahoo.com

**Keywords:** biomarkers, atherosclerosis, prognosis, development, research, midkine, pentraxins, inflammasomes

## Abstract

The atherosclerotic vascular disease is a cardiovascular continuum in which the main role is attributed to atherosclerosis, from its appearance to its associated complications. The increasing prevalence of cardiovascular risk factors, population ageing, and burden on both the economy and the healthcare system have led to the development of new diagnostic and therapeutic strategies in the field. The better understanding or discovery of new pathophysiological mechanisms and molecules modulating various signaling pathways involved in atherosclerosis have led to the development of potential new biomarkers, with key role in early, subclinical diagnosis. The evolution of technological processes in medicine has shifted the attention of researchers from the profiling of classical risk factors to the identification of new biomarkers such as midregional pro-adrenomedullin, midkine, stromelysin-2, pentraxin 3, inflammasomes, or endothelial cell-derived extracellular vesicles. These molecules are seen as future therapeutic targets associated with decreased morbidity and mortality through early diagnosis of atherosclerotic lesions and future research directions.

## 1. Atherosclerotic Vascular Disease—A Cardiovascular Continuum

**Figure 1:**
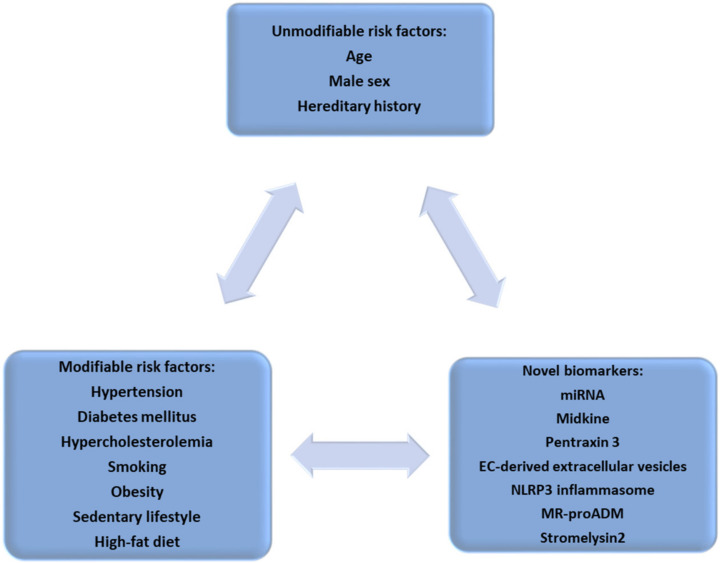
Risk factors associated with the occurrence of atherosclerotic vascular disease (EC: endothelial cell; MR-proADM: midregional pro-adrenomedullin; NLRP3: NOD-like receptor protein 3).

Atherosclerosis is a multifactorial process, in which genetic, environmental, and classical cardiovascular risk factors underlie complex pathophysiological processes that determine endothelial dysfunction and subsequent plaque formation in the vascular wall. Atherosclerosis is a chronic inflammatory disease with global economic and medical importance, with an increasing prevalence in the context of the continuing development of society [1,2]. Atherosclerosis is the basis of the cardiovascular continuum which includes a variety of cardiovascular diseases (CVDs) such as myocardial infarction (MI), aortic aneurysm, peripheral vascular disease, or stroke. Given that CVD is the leading cause of mortality and morbidity in industrialized countries and is increasing in developing regions of the world, the identification of new biological markers is a priority to enable the development of new therapeutic targets, as well as to facilitate the diagnostic algorithm for preclinical atherosclerosis [3,4] (Figure 1). Progression of atherosclerotic lesions occurs over time, and the removal of modifiable risk factors is essential for a favorable prognosis.

**Figure 2:**
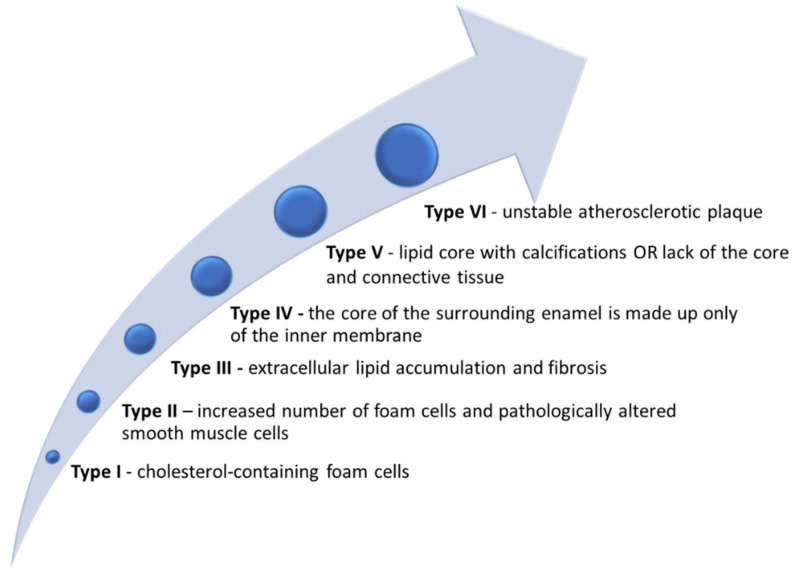
Atherosclerotic lesions: histopathological classification, role of lipid accumulation, involved cells, and matrix components in the progression of the atherosclerotic process.

From 1856 until present, significant progress has been made in understanding the mechanisms underlying the development and progression of atherosclerotic processes. Therefore, the discovery of biomarkers that allow early detection, thus preventing progression to complications accompanied by a high morbidity and mortality rate, has therapeutic and prognostic value alike. Rudolf Virchow was the first scientist to demonstrate, in 1856, that atherosclerotic lesions have extremely high lipid content (especially cholesterol and triglycerides) [5], which was further demonstrated by Nikolai Anichkov in 1913 in a study on rabbits [6,7]. Endothelial dysfunction, vascular cell activation, hypercoagulability, oxidative stress, and inflammation [8,9] are the main pathophysiological processes underlying atherosclerotic lesions. Extracellular matrix metalloproteinases (MMPs), macrophages, monocytes, proinflammatory cytokines, and apoB100/apoE receptors modulate chronic complex processes that support the concept of systemic, multifactorial pathology [10]. Six types of atherosclerotic lesions have been identified so far (Figure 2), of variable consistency, predisposing to the initiation or amplification of various signaling pathways [11]. Identification of type I–III lesions is characteristic for the early phase of the atherosclerotic process (the reversible one) compared to types V and VI, suggesting the presence of irreversible lesions [12,13]. 

## 2. Biomarkers—Pathophysiological and Clinical Implications—An Arch in Time

**Figure 3:**
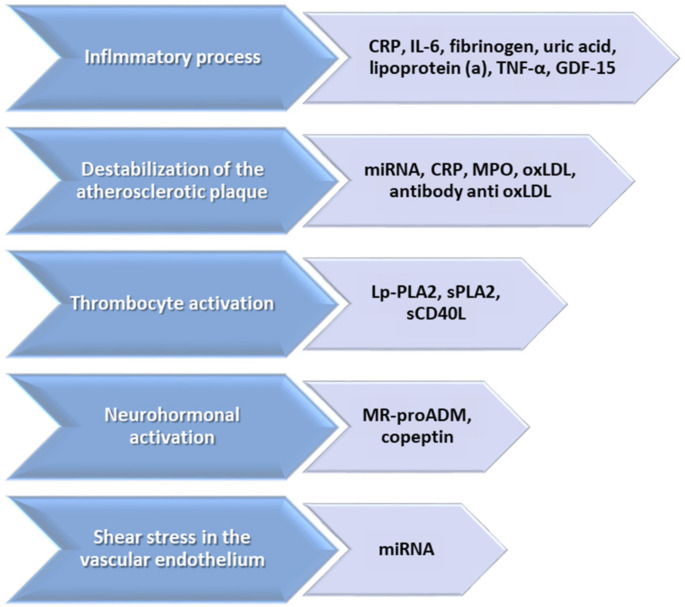
Biomarkers involved in the development and progression of atherosclerotic process (adapted after [12]) (CRP: C reactive protein; TNF: tumor necrosis factor; GDF-15: growth/differentiation factor-15; MPO: myeloperoxidase; oxLDL: oxidized low-density lipoprotein; Lp-PLA2: lipoprotein-associated phospholipase A2; Spla2: phospholipase A2; sCD40L: soluble CD40-ligand; MR-proADM: midregional pro-adrenomedullin).

The concept of biomarker was introduced in 1980 [14,15], representing a biological parameter with the role of objectively quantifying different indicators of normal biologic processes, pathogenic processes, or pharmacologic responses to a therapeutic intervention [16,17]. High sensitivity, high repeatability of results, and an economically or logistically easy clinical applicability represent the ideal characteristics of a biomarker [18]. The use of classical biomarkers represented by LDL-cholesterol, HDL-cholesterol, or serum triglycerides is only useful in patients with a high or very high cardiovascular risk, which has led over time to the discovery of molecules specific to the stages of the atherosclerotic process [19,20] (Figure 3).

Atherosclerosis is known to be a chronic inflammatory process in which interleukin 6 (IL-6), myeloperoxidase (MPO), matrix metalloproteinase 9 (MMP-9), and intercellular and vascular cell adhesion molecules are used as biomarkers. IL-6 and tumor necrosis factor α (TNF-α) [21] are two proinflammatory cytokines associated with increased cardiovascular risk and atherosclerotic plaque formation [22,23,24]. In patients with Type 2 diabetes mellitus, increased serum levels of IL-6 are associated with a higher failure rate of interventional revascularization [25]. MPO participates in the oxidation of LDL lipoproteins (oxLDL) which are highly atherogenic components. In addition to mediating the inflammatory process and destabilizing atherosclerotic plaques, MPO is also involved in mediating antibacterial and antiviral processes, which supports the multifactorial etiology of peripheral vascular disease [26]. Intercellular adhesion molecule 1 (ICAM-1) and vascular cell adhesion protein 1 (VCAM-1) are present in high concentrations in vessels prone to atherosclerotic processes, suggesting the presence of subclinical lesions [27,28]. C-reactive protein (CRP), fibrinogen, uric acid, and lipoprotein (a) (Lp <a>) are cardiovascular risk factors or biomarkers with multiple prognostic and therapeutic implications alike [12,29,30,31]. The role of malondialdehyde (MDA)-modified LDL-cholesterol as biomarker for atherosclerosis has been investigated in various studies so far [32]. Oxidized MDA-modified LDL are found in increased amounts in the blood as a result of the presence of active oxygen species and organic free radical generation [33]. Viigimaa et al. [33] demonstrated that MDA-modified LDL can be used as an independent biomarker for atherosclerosis, as it positively correlates with postinfarct cardiosclerosis (*p* < 0.05). In a similar study, the potential role of MDA in the progression of atherosclerotic lesions has been highlighted by Alghazeer et al. [34], who concluded that MDA levels indicate an increased production of oxygen free radicals. MDA correlates with the atherogenic index (*p* < 0.05) [32], the presence of oxidative stress being a risk factor associated with atherosclerosis which accentuates the need for antioxidant counterbalancing in dyslipidemic patients [35]. 

## 3. Midkine—A Biomarker for All Stages of Atherosclerosis

Midkine (MK) is a heparin-binding growth factor, part of the family of growth factors and cytokines. MK mediates various cellular processes, being involved in both inflammatory and reparative processes, as well as in oncogenesis [36,37]. MK is localized preferentially at the level of advanced atherosclerotic plaque in the thickened intima of fatty streaks, forming, with vascular smooth muscle cells, extracellular cells, and inflammatory agents, a dense and diverse cellular beach with a proinflammatory role that ensures the progression of the atherosclerotic process [38].

MK has a pro-atherogenic effect and is involved in multiple stages of the atherosclerotic process, such as lipid accumulation, inflammation, or neointima formation [37,38]. MK stimulates macrophage lipid accumulation and may be considered a promoter of dyslipidemia. MK is found in vascular smooth muscle cells (VSMC), endothelial cells, and inflammatory cells. Between MK and serum levels of total cholesterol or LDL-cholesterol, there is a directly proportional relationship, independent of the presence of hypertension [39]. Mutations of the ATP binding cassette transporter A1 (ABCA1), a transmembrane protein involved in the intracellular cholesterol efflux to lipid-poor apolipoprotein A-I, leads to premature atherosclerosis. Ou et al. [40] demonstrated that it downregulates ABCA1 expression by inhibiting the phosphorylation of AMP-activated protein kinase. This effect, also evidenced by other clinical studies, raises the hypothesis that blockade of cholesterol efflux via ABCA may lead to the arrest of the atherosclerotic process, being a future research direction in the pharmacological field of dyslipidemia. VSMC can turn into foam cells and can be found in large amounts in intimal plaques at all stages of the atherosclerotic process [41]. VSMC senescence promotes atherosclerosis [42] and increases the vulnerability of the plaques [43]. Independent of the stage, MK is assumed to have a modulatory role, related to the negative regulation of ABCA1 expression [44,45]. MK also plays an essential role in inflammatory processes in atherosclerotic plaques, promoting T cell activation and Th1 cell differentiation [46] and increasing mRNA levels of proinflammatory cytokines such as y interleukin-1β and interferon-γ [37]. Clinical studies have also shown that MK promotes monocyte attachment to the arterial wall for differentiation into macrophages. Kosugi et al. [47] previously concluded that MK downregulates monocyte chemoattractant protein-1 expression. Consistent with previous findings, Takemoto et al. [37] demonstrated in an vitro study that administration of MK is associated with increased serum levels and regulates monocyte chemoattractant protein-1 in aortic tissues which leads to the accumulation of macrophages in atherosclerotic lesions [48,49]. MK also plays an essential role in apoptosis by inhibiting it in cardiomyocytes. Macrophage apoptosis inhibits the progression of atherosclerotic processes, its prevention being one of the mechanisms by which MK exerts its atherogenic effect [50,51]. MK is an important factor in patients with advanced atherosclerosis and may be one of the missing links in the pathomechanism of this disease. Serum MK levels are elevated in the circulation of both adult and pediatric patients with heart failure and positively correlate with the severity of the clinical picture [52,53]. In patients with coronary artery disease, the administration of MK was associated with improved survival and decreased infarct size as a result of stimulation of angiogenesis and inhibition of myocyte apoptosis [54]. On the other hand, cardiac hypertrophy is associated with increased expression of MK in myocytes with a stimulatory effect on fibrosis [54,55,56]. 

Just as MK is expressed in cardiomyocytes under ischemic conditions and overexpressed after myocardial infarction, the same mechanism is probably involved in peripheral ischemia, with higher MK values in acute ischemia. Of particular interest is the inverse association with antibody-induced signaling against angiotensin 1 receptor (AT1R) and endothelin A receptor (ETAR) [57]. MK induces angiotensin-converting enzyme (ACE) expression in microvascular endothelial cells as a regulator of the renin–angiotensin system (RAS). Significantly lower serum MK levels were found in cardiac transplant patients treated with ACE-inhibitors or angiotensin-receptor blockers (ARB). The same mechanism could underlie for AT1R and ETAR antibodies, which are inversely correlated with MK levels. The corroborating data bring together the renin–angiotensin system and the endothelin system as hallmark effectors in atherosclerosis, and a mediator of angiogenesis. A possible explanation for this phenomenon would be the “inner balance” of the atherosclerotic process as a multifactorial disease. The more important the general state of inflammation, angiogenesis, and proliferation in the vascular wall, represented by high levels of MK, the lower the activation of the immune system involved in athero-progression, in this case represented by functional autoantibodies against well-known effectors of the atherogenic process. Guzel et al. demonstrated that MK serum levels correlate positively with hypertension and lipid profile [39]. Besides MK, clinical studies have also revealed other biomarkers associated with CVD, such as heart failure and atherosclerosis such as midregional pro-adrenomedullin or stromelysin2 studied in isolation or associated with MK [58]. 

## 4. Biomarkers of Destabilization of Atherosclerotic Plaque—microRNA

MicroRNAs (miRNAs) are small single-stranded RNA molecules that mediate multiple biological processes that contribute to the development and progression of atherosclerotic processes such as angiogenesis, apoptosis, or cell differentiation [59]. The structure of these molecules is complex, with coding genes located in genetically unstable regions in the introns or exons of protein-coding genes as well as in intergenic regions [60,61]. miRNA regulate up to 60% of the genes, and mutations or aberrant expression patterns are associated with the occurrence of various diseases such as CVD, infections, neurodegenerative diseases, or cancer (through tumor-induced suppression) [62,63]. Extracellular miRNAs have similar blood serum levels among healthy people. In addition to blood tests, they can be detected in the body by analyzing various fluids such as urine, bronchial lavage, synovial fluid, breast milk, saliva, and cerebrospinal fluid [64,65]. Their ease of dosing promotes them as potential biomarkers that allow early diagnosis and also as next-generation therapeutic targets.

miRNAs and pre-miRNAs are found in the extracellular environment in free (stable) form, either embedded in circulating microvesicles, exosomes, high-density lipoprotein (HDL), or protein complexes. Through their associated properties, these molecules mediate various cellular processes in atherosclerotic plaques, thus contributing to their progression [66]. The role of HDL in the transport of miRNAs has been previously demonstrated by Vickers et al. [67]. The molecular analysis of the HDL–miRNA complex revealed differences between apparently healthy subjects and those with familial hypercholesterolemia. In addition, in patients with atherosclerotic lesions, a distinct gene induction was observed, associated with the loss of conserved mRNA targets in cultured hepatocytes.

miRNAs regulate lipoprotein metabolism and ensure a balance between atherogenic low-density lipoproteins (LDL) and the atheroprotective ones [68]. miRNAs decrease cholesterol absorption in the liver by inhibiting the expression of cholesterol transporters and of the scavenger receptor BI (SR-BI), as well as the LDL receptor. By modulating cholesterol homeostasis, these biomarkers intervene in the early stages of the atherosclerotic process [69]. Among all the molecules included in the miRNA family, miR-122 is an essential building block through its demonstrated role in modulating cholesterol and fatty acid synthesis in the liver, highlighting it as a future target for the treatment of dyslipidemia [70]. 

miRNAs are involved in all stages of the atherosclerotic process, from its initiation to the mediation of processes involved in neoangiogenesis. Endothelial dysfunction might be considered as the starting point of the atherosclerotic process, leading to a proinflammatory status through the activation of proinflammatory cytokines and oxidative stress [66]. A number of molecules, such as tumor necrosis factor α (TNF-α), angiotensin II, or oxidized (ox) LDL, cause increased cellular expression of endothelial leukocyte adhesion molecules (such as E-selectin or VCAM-1) and intracellular adhesion molecule (ICAM)-1 [71,72]. The role of miRNAs in modulating inflammatory processes has been demonstrated in multiple clinical studies, the main representatives involved being miR-181b, miR-181a-5p, and miR-181a-3p [73]. miR-181a-5p and miR-181a-3p have an anti-inflammatory effect by inhibiting the expression of VCAM-1, ICAM-1, and E-selectin. The same effect was also observed in the case of miR-181b, which inhibits gene expression via the importin-3 protein, influenced by NFkB nuclear translocation [72].

The interaction between miRNAs and microvesicles has been demonstrated in various in vitro studies, in particular from the perspective of their active role in apoptosis, inflammation, and cell proliferation, central pathophysiological mechanisms of endothelial dysfunction [71,74,75]. The mechanism by which miRNAs are incorporated into microvesicles is not fully known so far, being presumed an ATP-dependent process influenced by the extracellular environment [76,77]. The prospective role of these molecules in the early diagnosis of atherosclerosis has also been demonstrated by Jansen et al. in a study in which 176 patients with coronary artery disease were enrolled. They demonstrated that miR-126 or miR-199a expression levels in circulating microvesicles were positively correlated with recurrence of an acute cardiovascular event, compared to plasma doses [71,78]. 

Oxidative stress and nitric oxide bioavailability are also mediated by various molecules in the miRNA family. Thus, oxidative stress increases gene expression of miR-200c and miR-199a-3p. miR-199a-5p and miR-199a-3p mediate nitric oxide bioavailability by increasing endothelial NO synthase (eNOS) activity. eNOS activity is also modulated by various enzymes, such as superoxide dismutase (SOD)1 and peroxiredoxin (PRDX)1, which indirectly determines the upregulation of phosphatidylinositol 3-kinase (PI3K)/protein kinase B (Akt) and calcineurin pathways [79,80,81]. Endothelial cell senescence contributes to the progression of atherosclerotic lesions; further study of the mechanisms and the role of miRNAs in this process are possible targets for reversing or diminishing the aging-associated effect. Clinical studies to date have demonstrated that miR-216a is involved in the process of premature aging of endothelial cells, its effect being exerted on the signaling pathway mediated by the transforming growth factor (TGF)-β1 [82,83]. 

In addition to endothelial dysfunction, miRNA family molecules are involved in other processes in atherosclerotic plaques such as monocyte recruitment, macrophage differentiation, or foam cell formation. Macrophages secrete extracellular vesicles containing various molecules of the miRNAs family, with the most highly expressed secondary to oxLDL exposure being miR-146a. This prevents macrophage migration from atherosclerotic plaques by inhibiting the insulin such as growth factor 2 mRNA binding protein 1 (IGF2BP1) and human antigen R (HuR) [74,84,85]. miR-21 exerts its effect in the early stages of atherosclerosis, acting in particular on vascular smooth muscle cells (VSMCs) by increasing the proliferative capacity and the synthesizing phenotype. In addition to miR-21, the modulatory role of miR-1 on contractile proteins in VSMCs has also been demonstrated, leading to suppression of α-smooth muscle actin [86,87,88].

miRNAs are also involved in atherosclerotic plaque rupture through acting on inflammatory cells and on the necrotic core covered by a thin fibrous cap [89]. Jin et al. demonstrated that miR-21 overexpression inhibits ROS-induced smooth muscular cell apoptosis in vitro [90]. In a similar study, Jin et al. concluded that local administration of miR-21 to carotid atherosclerotic plaques increases plaque stability secondary to smooth muscular cell proliferation [90,91]. Molecules from the miRNA family contribute to the formation and expansion of vasa vasorum in the atherosclerotic lesions [92,93,94]. miRNAs are regulators of the VSMC and induce their overgrowth and transformation [95]. 

The expression of the various molecules of the miRNA family differs according to the stage of atherosclerotic lesions, the main biomarkers correlated with the occurrence and progression of CVD being miR-21, miR-92a and miR-99a [96]. Several miRNAs have been found in different stages of atherosclerosis disease: miRNA-10a/b, miRNA-17-3p, miRNA-31, and miRNA 126 are found in endothelial cells, miRNA-33a/b and miRNA-122 are involved in cholesterol homeostasis, miRNA-21, miRNA-26a, miRNA-155, and miRNA-221 contribute to plaque development, miRNA-27a/b, miRNA-155, miRNA-210, miRNA-221, and miRNA-22 are markers of neoangiogenesis, and miRNA-100, 127, 133a/b, and 145 are suggestive of the presence of vulnerable plaque [97].

**Figure 4:**
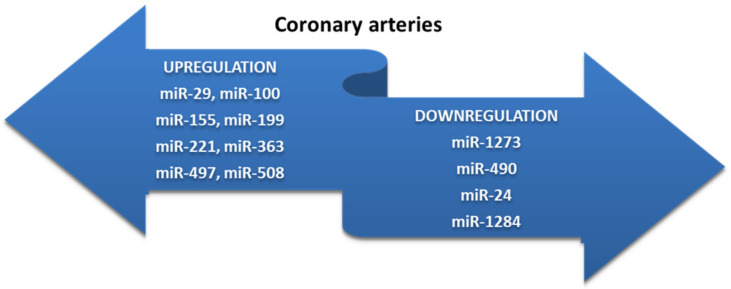
miRNAs as biomarkers in coronary artery disease (adapted from [116]).

**Figure 5:**
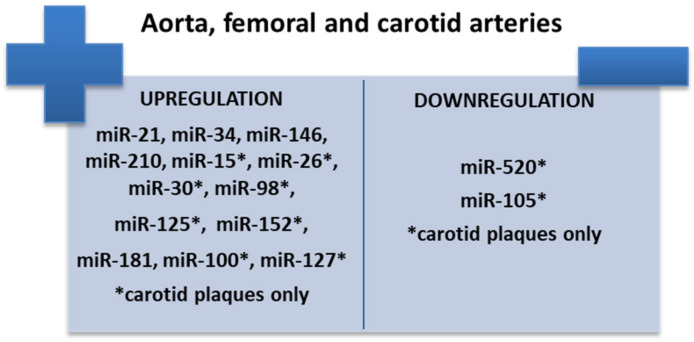
miRNAs as biomarkers in peripheral artery disease (adapted from [116]).

Clinical studies to date have demonstrated that miR-21 is accompanied by elevated serum levels in patients with clinical manifestations. Differences in gene expression of the molecules have also been targeted according to the location of atherosclerotic lesions. Thus, carotid atherosclerotic plaques are accompanied by elevated serum levels of miR-21 and miR-29 (Figure 4). The presence of atherosclerotic lesions in the lower limbs results in reduced gene expression of miR-27b, miR-130a, and miR-210 [98,99,100,101] (Figure 5). Patients with coronary artery disease (CAD) associate low serum levels of miRNAs secondary to their sequestration in atherosclerotic plaques, which mediate neoformation processes and maintain proinflammatory status [102]. Special attention has also been paid in recent studies to noncoding RNAs (ncRNAs) which are involved in several processes such as atherosclerosis, angiogenesis, neuroinflammation, or apoptosis, but their clinical applicability is currently limited [103,104,105]. Zhao et al. [103] concluded that ncRNAs are potential biomarkers for early diagnosis of ischemic stroke similar to miR-107 and miR-135b [106,107]. Type 2 diabetes mellitus is a risk factor for the development and progression of CAD, changes in serum levels of various miRNAs can be used for diagnosis and prognostic stratification [108,109,110]. Several miRNAs, such as miR-1, miR-122, miR-132, and miR-133, are associated with the development of subclinical atherosclerotic lesions in patients with metabolic syndrome [111]. There are a number of differences between the time of serum level change and clinical significance. Thus, while miR-1, miR-132, miR-133, and miR-373-3-p suggest the concomitant presence of CAD and Type 2 diabetes, the increase in serum miR-92a levels in diabetic patients occurs independently of coronary artery involvement [112,113]. Furthermore, miR-122-5p and miR-223-3p are upregulated in patients with unstable CAD and may be considered a marker of atherosclerotic plaque vulnerability [114,115]. Not all molecules in the miRNA family have elevated serum levels, with clinical studies showing a downregulation effect in miR-1273, miR-490, miR-24, and miR-1284 [116]. miR-32 is also a promising biomarker in CAD patients, with high levels of miR-32-5p identified in their exosomes [110,117,118]. MK is not the only biomarker that modulates ABCA1 activity; changes in its genetic structure are secondary to post-transcriptional regulation mediated by miRNAs [119]. 

Wang et al. demonstrated the existence of an inversely proportional relationship between serum levels of miRNAs and severity of coronary atherosclerotic damage [66,101]. miRNA-155 upregulation after the occurrence of a myocardial infarction has been demonstrated by Schumacher et al. The investigators conclude that miRNA-155 is associated with cardiac remodeling via inflammation and fibroblast recruitment in these patients [120]. In a dyslipidemic mouse model, the investigators concluded that although genetic depletion of miRNA-155 decreased myofibroblast density in the post-ischemic scar, it has no impact on the infarction size [120]. Li et al. demonstrated the cytoprotective role of miRNA-144 in an ischemia/reperfusion injury model on mice. At 28 days follow-up after intravenous administration of miRNA-144, the investigators observed that infarct size decreased and left ventricular contractile function improved compared to the control group. Reduction of fibrosis area, anti-inflammatory role, and reduction of apoptosis were some of the positive effects observed, which raised the hypothesis of the development of miRNA-144 targeted therapeutic agents and the need for further clinical trials to validate the data [121].

## 5. Pentraxin 3

Pentraxins are acute phase proteins that mediate various cellular processes or pathophysiological mechanisms such as inflammation, angiogenesis, cell adhesion, or neoplasia [122]. These molecules are well known for their role in humoral innate immunity. Depending on the length of the N-terminal region, they are divided into two categories: small ones, such as C-reactive protein (CRP) [123,124], which are synthesized in the liver secondary to the action of IL-6, and long constituents, such as pentraxin 3 (PTX3), which are produced by vascular and immune cells secondary to the proinflammatory status and through the involvement of toll-like receptors [125,126]. PTX mediates its effects by interacting with numerous ligands such as microorganisms, plasma proteins, extracellular matrix constituents, receptors, or growth factors. Their role in mediating inflammatory processes and interaction with serum complement and immune system components explains their role in the atherosclerotic process. 

PTX3 is a representative of the class involved in the emergence and progression of CVD. PTX3 modulates inflammatory processes by inhibiting leukocyte recruitment [127,128]. PTX3 is highly expressed in smooth muscular cells through atherogenic lipoproteins, with clinical studies showing elevated levels in advanced stages of the atherosclerotic process. PTX3 determines upregulations of the tissue factor expression in endothelial cells and activated monocytes [129,130,131]. PTX3 modulates endothelial dysfunction by its direct effect on endothelial cells. Serum PTX3 levels are mediated by the action of excessively produced IL-1β and TNF-α in patients with a systemic inflammatory stress [132,133].

The role of PTX3 as a marker of inflammatory status and prognostic biomarker in CVD has been demonstrated in multiple clinical trials in patients with or without clinical CAD. Jenny et al. demonstrated that PTX3 is an independent prognostic factor reflecting the risk of CVD (odds ratio 1.11) and all-cause death (odds ratio 1.08), independent of serum CRP levels or the presence of cardiovascular risk factors [134]. Furthermore, patients with subclinical atherosclerotic lesions were associated with higher serum PTX3 levels compared to those without atherosclerotic lesions (*p* = 0.001). The same investigators demonstrated 5 years later that increased serum levels of PTX3 positively correlate with age, obesity, systolic blood pressure, serum CRP levels, or carotid intima-media thickness (*p* values below 0.045 for all parameters) [135]. The lack of correlation between serum levels of PTX3 and CRP raises the hypothesis of differential mediation of the inflammatory process in atherosclerotic plaques, but further studies are needed in this regard. There is conflicting evidence in the literature that PTX3 and CRP may have an antagonistic effect in the development of metabolic syndrome and obesity [136,137]. While CRP correlates with body weight, body mass index, waist circumference, fasting plasma glucose, or IL-6, PTX3 levels associate with adiponectin, but not with the molecules or constituents mentioned above. Ogawa et al. concluded that PTX3 levels were lower and CRP levels were higher in patients with more than one component of the metabolic syndrome compared to apparently healthy patients [136]. PTX3 is considered to be a valid biomarker candidate for atherosclerosis due to high plasma levels observed in patients with carotid stenosis or MI. In addition to lesion severity, PTX3 can also assess plaque vulnerability and is considered a marker of vascular injury and subsequent neoangiogenesis [138,139,140,141].

HDL stimulates gene expression of PTX3 in human umbilical vein and in the endothelial aortic cells, suggesting a link between decreased serum HDL levels and decreased PTX3 levels. Salio et al. suggest cardioprotective and anti-inflammatory effect of PTX3 in a mouse-model-induced acute myocardial infarction [142,143]. Nauta et al. [144] highlight that PTX3 can both activate and inhibit the complement-mediated signaling pathway, suggesting that decreased PTX3 levels in patients with metabolic syndrome cause progression of the atherosclerotic process by maintaining the chronic inflammatory status. Carrizzo et al. demonstrated that PTX3 administration in mice causes endothelial dysfunction and increased blood pressure via the P-selectin/MMP1, which then acts through nitric-oxide-mediated signaling pathways [145,146].

PTX3 is highly expressed in the heart, and in patients with acute MI, the maximum serum level is reached 7 h after the occurrence of the cardiac event. Latini et al. [147] demonstrated that in patients with MI with ST elevation, compared to CRP or NT-proBNP, TnT, or creatin kinase, PTX3 was the only biomarker able to predict mortality risk 3 months after the event. PTX3 has an inhibitory effect on neoangiogenesis, VSMC proliferation, parietal thickening, or restenosis, effects mediated mainly by the increased ability of this molecule to bind fibroblast growing-factor 2 (FGF2) [125,148,149]. Increased levels of PTX3 correlate with CAD severity and negatively influence the prognosis of elderly patients [150]. PTX3 is also a biomarker of the diabetic vasculopathy, with elevated serum levels being identified in carotid and coronary plaques in patients with acute myocardial infarction [25,137,151]. 

**Figure 6:**
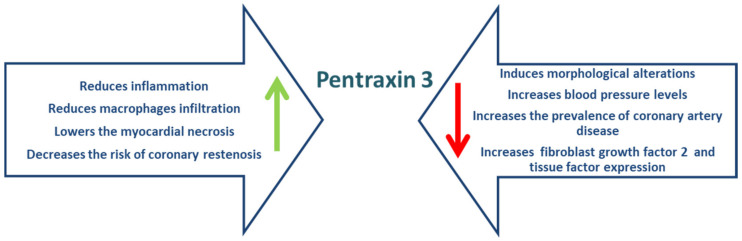
Effects of pentraxin 3 (adapted after [150]).

PTX3 has demonstrated its role as a biomarker in both CAD and heart failure [141]. Akgul et al. [152] concluded that high levels of PTX3 at admission lead to an increased mortality rate both in hospital and 2 years after the acute event, making PTX3 an independent predictor. In a prospective observational study on 75 patients with MI, Kimura et al. demonstrated that the presence of a high level of PTX3 (above 3.79 ng/mL) in atherosclerotic plaques prior to PCI is associated with a high rate of rupture or coronary artery bypass grafting. In a similar study [153], Guo et al. observed a correlation between PTX3 levels above 3 ng/mL and elevated serum high sensitivity CRP (hs-CRP), cTnT, NT-proBNP, and the coronary stenosis degree [154]. In patients with heart failure, PTX3 correlates with hospitalization rate, the occurrence of acute cardiovascular events, or cardiac death [155,156]. In conclusion, PTX3 has both a beneficial and a deleterious effect, the balance between atheroprotective and proinflammatory effects being a challenge for research in the development of a future therapeutic strategy focused on the action of this biomarker (Figure 6).

## 6. Endothelial-Cell-Derived Extracellular Vesicles 

Extracellular vesicles (EVs) represent a heterogeneous family of vesicles secreted by various cells, and by which their diverse biological functions mediate multiple physiological and pathophysiological processes [157,158]. Their active role in the progression of atherosclerotic processes has been demonstrated in multiple studies, as these cell organelles are involved in various cell signaling pathways such as vascular remodeling, inflammation, or cell proliferation and migration [159,160]. The activation of extracellular cells can be seen as the starting point of the atherosclerotic process, endothelial dysfunction playing an essential role in the production of EVs in endothelial cells [161]. EVs have a transport role by incorporating various substances such as cholesterol or genetic material (RNA or small noncoding RNAs) [162,163]. Clinical studies have demonstrated the active role of EVs both in the early stages of the atherosclerotic process and in complicated lesions [164,165]. EV levels correlate with the presence of risk factors, high levels being identified among patients who smoke, have dyslipidemia, or have high blood pressure [166,167,168,169,170]. 

EVs can be seen as both liquid biomarkers and potential therapeutic vectors in CVD [171,172]. Clinical studies show a positive correlation between elevated blood, saliva, or urine levels and the risk of a cardiovascular event in patients with stable CAD [173,174,175]. 

EVs located in highly thrombogenic atherosclerotic plaques originate from various cells, the most common being leukocytes, macrophages, and erythrocytes [176,177]. EVs mediate the lipid streak formation process by increasing gene expression of endothelial molecule and suppression of NO synthesis, as well as macrophage migration. EVs derived from monocytes promote inflammatory processes and upregulation of ICAM-1, VCAM-1, and E-selectin, as well as vascular cell death. EVs contribute to the destabilization of atherosclerotic plaques by degradation of the extracellular matrix, which leads to the slimming of the fibrous cap and secondly to plaque rupture [178,179,180,181]. EVs derived from endothelial and platelet cells are the main organelles found in the blood [78,173]. Nozaki et al. [173] demonstrated that measuring plasma levels of endothelium-derived microparticles can be seen as an indirect way of assessing endothelial dysfunction, as well as being a statistically significant prognostic factor (odds ratio 1.042, *p* = 0.02) in the prediction of acute cardiovascular events in high-risk patients (assessed on the basis of the Framingham risk score). The EVs secreted by foam cells, endothelial cells, or platelets modulate macrophage activity, having an active role in the progression of atherosclerotic lesions by inhibiting vasorelaxation and maintaining vascular inflammatory status [182].

Cellular senescence is associated with a change in the secretory profile with activation of immune cells and the appearance of a chronic systemic inflammatory status [183]. The release of an increased amount of EVs alters paracrine signaling pathways while also influencing the metabolism of neighboring cells, leading to endothelial dysfunction [184]. The senescence-associated secretory phenotype is characterized by increased levels of growth factors, decreased DNA replication, and increased concentrations of proinflammatory markers and matrix metalloproteinases [185]. Senescent endothelial cells have an increased concentration of vasoconstrictor molecules (reduction of nitric oxide, increase of ROS), thrombotic factors (tissue factor and plasminogen activator inhibitor-1), and adhesion molecules [183,186]. 

EVs are involved in the calcification process of atherosclerotic lesions by increasing calcium and bone morphogenetic protein 2 (BMP-2) levels, thereby promoting the osteogenic phenotype of the smooth muscle cells [187]. Reduced collagen percentage at the fibrous cap level weakens the structure and makes it prone to rupture [188]. EVs’ role in plaque destabilization and thrombus formation has been highlighted in multiple clinical studies. EVs at the level of vulnerable plaques have a high content of thrombogenic microvesicles capable of generating tissue factor and thrombin, which justifies the associated procoagulant potential [165,176].

Based on the idea that EVs mainly carry miRNAs, the validation of a risk score based on the association of the two biomarkers is a topic of interest in the field. Jansen et al. demonstrated that elevated levels of EV miR-126 and miR-199a correlate with a lower adverse rate of cardiovascular events in patients with stable CAD, an aspect that is not evident in the case of the solitary evaluation of soluble miRNAs [78,157]. In a similar study, Goetzl et al. concluded that protein content analysis of EVs correlates with progression of atherosclerotic lesions [189]. As mentioned before, several biomarkers are involved in the diagnosis, management, and treatment of diabetic vasculopathy, including exosomes [190]. Exosomes induce changes in cellular metabolism both locally and remotely by mediators released into the blood, urine, or tears, thus being useful biomarkers [191]. Increasing attention is being paid to stem cells exosomes as a promising therapeutic target in the treatment of diabetic vascular complications. Although they were initially considered as cell replacement therapy, subsequent clinical studies have demonstrated that their action is mainly based on the release of mediators that stimulate tissue repair [190,192,193].

The role of exosomes as potential therapeutic targets has been demonstrated in various mouse models based on their ability to incorporate various molecules with therapeutic value such as miRNAs or mRNA vectors. Exosomes have multiple benefits in terms of carrier role and associated biocompatibility, but they also have a number of associated limitations due to their quantitative efficiency [177,194]. These clinical studies highlight the potential role of biomarkers in the assessment of atherosclerotic lesions, but further studies are needed to validate the associated prognostic risk.

## 7. Role of NLRP3 Inflammasome in Atherosclerosis

Atherosclerosis is a complex process, mediated in part by and through the innate immune system. Inflammasomes are an essential component involved in the development, progression, and rupture of atherosclerotic plaques [195]. These constituents were first described in 2002 by Schroder and Tschopp [196] by highlighting their role in caspase-1 activation and interleukin-1β processing. The inflammasome containing the nucleotide-binding oligomerization domain, leucine-rich repeat-containing receptor (NLR) family, and pyrin domain-containing 3 (NLRP3) has been the best studied to date and plays an essential role in the initiation and progression of atherosclerotic processes and the mediation of vascular inflammation [197]. NLRP3 inflammasomes are mainly involved in inflammatory processes in atherosclerotic lesions [198]. Recent studies have focused attention on the potential therapeutic role of NLRP3. Components of NLPR3 have been identified in macrophages and fat cells of carotid atherosclerotic plaques [199]. Extracellular cells also contain NLPR3 [200]. Zheng et al. demonstrated the existence of high levels of NLPR3 inflammasome constituents in atherosclerotic plaques [201]. Activation of NLPR3 generates a trigger signal for activation of caspase-1 which in turn induces the production of interleukin-1β (IL-1β) and interleukin-18 [202]. IL-1β plays an essential role in neoangiogenesis, mediating both cellular processes in circulating cells and in the arterial wall [203]. Menu et al. [204] demonstrated that genetic depletion of interleukin-1β or interleukin-1 receptor inhibits the progression of atherosclerotic lesions in hypercholesterolemic mice [205]. 

Hypoxia, oxLDL, atheroprone flow, neutrophil extracellular traps, or somatic mutations are the main factors that activate NLRP3 inflammasome [206]. Murine plaques contain multiple hypoxic regions which cause angiogenesis, stimulating foam cell or plaque necrotic core formation [202,207]. Consistent with the above, Folco et al. [208] demonstrated that hypoxia increases NLRP3 expression and stimulates caspase-1 activation in cultured human macrophages which subsequently secrete large amounts of IL-1β contributing to the maintenance of the proinflammatory status associated with atherosclerosis. Shear stress contributes as a trigger for NLRP3 inflammasome activation in atherosclerotic lesions [200]. NLRP3 inflammasome activation interferes with lipid metabolism, stimulating macrophage migration capacity and lipid particle uptake into lysosomes in macrophages [209]. Accumulation of macrophages in the arterial wall stimulates formation of foam cells that contribute to the progression of atherosclerosis. Activation of the NLRP3 inflammasome triggers pyroptosis and release of inflammatory substances factors contributing to the maintenance of proinflammatory status [210]. The therapeutic role of IL-1β was demonstrated in the CANTOS (Canakinumab Anti-inflammatory Thrombosis Outcomes Study) clinical trial [211] in which patients were treated with canakinumab (a human monoclonal antibody which binds IL-1β) associated with decreases in serum levels of inflammatory markers (CRP) and were associated with a reduced risk of acute cardiovascular events compared to the placebo-treated group of patients. NLRP3 inflammasome modulates the IL-1 and IL-6 family cytokine production [212,213]. These results support the concept that NLRP3 inflammasome may be a therapeutic target, antagonizing its proinflammatory effect, contributing to the prevention of atherosclerosis. Arglabin inhibits the activation of NLRP3 inflammasome in macrophages, thus having an anti-inflammatory effect on atherosclerosis-prone mice by lowering serum levels of IL-1β [214]. NLRP3 inflammasomes are involved in cigarette-smoke-induced atherosclerosis [215]. Cigarette smoke activates NLRP3 inflammasome in monocytes, macrophages, and foam cells [216,217] Embolization of cholesterol crystals from atherosclerotic lesions of the major arteries causes the appearance of cholesterol-embolization syndrome, known as a systemic disease [218,219]. Its etiology is iatrogenic, mainly secondary to interventional or surgical procedures. The presence of cholesterol crystals leads to activation of pathophysiological pathways mediated by NLRP3 and IL1 [218]. 

NLRP3 inflammasome is a valuable constituent with both prognostic and therapeutic value and its targeting could be the basis for new therapeutic classes in the treatment of atherosclerosis [202,220]. 

## 8. Biomarkers of Thrombocyte Activation—Midregional Proadrenomedullin

Adrenomedullin (ADM) is a vasoactive peptide involved in multiple cardiovascular pathophysiological processes such as vasodilatation, natriuresis, increasing cardiac output, or modulation of vascular calcification [58]. Moreover, ADM inhibits VSMC proliferation and neoangiogenesis and promotes the re-epithelialization process [221]. The diagnostic and prognostic value of this molecule in patients with CAD or heart failure has been demonstrated in multiple clinical trials. The evolution of molecular diagnostic methods has allowed the identification and characterization of WMD precursors such as midregional pro-adrenomedullin (MR-proADM). MR-proADM is a more stable molecule whose serum levels allow prognostic assessment of patients with sepsis, systemic inflammation, or cardiac dysfunction [222,223].

MR-proADM midkine and stromelysin2 (ST2) are biomarkers associated with various CVDs such as heart failure or atherosclerosis. MR-proADM can also be used as a biomarker for arterial stiffness in patients with metabolic syndrome, its predictive value being superior to that of CRP [224]. 

The prognostic role of MR-proADM in patients with MI has been studied in several clinical trials so far. The identification of new biomarkers to allow early, preclinical diagnosis of atherosclerosis is an ongoing concern of the scientific society. The development and clinical validation of risk scores based on a range of biomarkers allow the development of diagnostic and treatment algorithms that, if applied promptly, can improve long-term prognosis. Melander et al. [225] evaluated the prognostic role of five biomarkers (CRP, midregional-pro-atrial natriuretic peptide, N-BNP, MR-proADM, Lp-PLA2, and cystatin C) in assessing cardiovascular risk. The investigators demonstrated, using regression analysis, the predictive value of N-BNP and CRP for cardiovascular events, while N-BNP and MR-proADM have proven effective in assessing the risk of acute coronary events. In a similar study, Ross et al. demonstrated that MR-proADM levels can predict the presence of a coronary artery stenosis (*p* = 0.026) or soft atherosclerotic plaques (*p* = 0.026) in patients without known CVD [226]. Yoshihara et al. evaluated the role of MR-proADM as a biomarker of cardiac dysfunction in hemodialysis patients and concluded that increased plasma levels correlate positively with cardiac dysfunction, inflammatory status, and with volemia in patients with concomitant renal and cardiovascular disease [227]. Clinical studies have demonstrated a positive relationship between elevated serum levels of MR-proADM and the occurrence of cardiovascular morbidity and mortality, raising the hypothesis of the usefulness of this marker, especially in young subjects [228]. Neumann et al. also pointed out the association of this molecule with classic cardiovascular risk factors and atherosclerotic disease. MR-proADM levels vary depending on age, the presence of hypertension or diabetes mellitus, dyslipidemia, atrial fibrillation, or presence of systemic atherosclerosis damage [229]. A number of gender-dependent differences were also observed, pointing to a statistically significant association between MR-proADM, hypertension, and echocardiographic parameters of diastolic dysfunction in men.

Besides MR-proADM, cystatin C and lipoprotein-associated phospholipase A2 are biomarkers involved in thrombocyte activation. Cystatin C is a cysteine protease inhibitor found in all tissues and body fluids [230]. It plays an important role in the atherosclerotic process, acting mainly at the level of the vascular wall by inhibiting the cathepsin-dependent proteolytic activity. Atherosclerotic plaques are associated with decreased cystatin C levels [231]. Nishimura et al. [232] recently demonstrated that preoperative serum levels of cystatin C can be used as a biomarker for aortic plaque in the descending aorta as it positively correlates with the total aortic plaque volume ratio. In a similar study, it was shown that cystatin C is associated with carotid thickening and plaque [233]. Cystatin C is also a useful tool for cardiovascular risk stratification. Correa et al. [234] demonstrated that cystatin C is a useful biomarker for risk assessment of adverse cardiovascular events after an acute coronary syndrome. 

Lipoprotein-associated phospholipase A2 (Lp-PLA2) plays an essential role in cardiovascular risk stratification [235,236]. Although accompanied by low blood bioavailability, high serum levels are associated with increased risk of acute cardiovascular events [237,238]. Lp-PLA2 reflects intravascular inflammations and the presence of unstable plaques [239]. Therapeutic efficacy of Lp-PLA2 inhibitors has been investigated, proving its beneficial role on necrotic core volume of coronary plaque. Still, administration of darapladib, an Lp-PLA2-inhibitor, did not reduce the rate of acute coronary events in several clinical trials [240,241,242]. Lp-PLA2 is not present in incipient atherosclerotic lesions, being identified in increased amounts in the thin fibrous cap of rupture-prone plaques [243,244,245]. Lp-PLA2 serum levels are independently correlated with CAD and it has a 53.0% sensitivity and 80.3% specificity for recognizing severe CAD lesions [246]. 

## 9. Stromelysin-2 

Matrix metalloproteinases (MMPs) are a family of endopeptidases involved in various cellular processes in atherosclerotic plaques, being responsible for their progression or complications (mainly rupture) [247]. Destabilization and rupture of atheroma plaques occurs secondary to an imbalance between MMPs and their inhibitors [248]. In addition to modulating atherosclerotic plaque processes, MMPs ensure the integrity and functionality of vessels as well as cardiovascular remodeling [249]. These molecules are synthesized by a variety of cells, the most commonly cited in the literature being endothelial cells, VSMC, fibroblasts, or macrophages [250,251]. 

Stromelysins are a subfamily of MMPs, the main representative being stromelysin-2, also known as MMP-10. MMP-10 has a proinflammatory role, being secreted by macrophages secondary to the presence of injury or inflammatory stimuli. Several studies have concluded so far that increased serum levels of proMMP-10 are associated with both clinical and subclinical atherosclerosis. MMP-10 are expressed and secreted by human atherosclerotic plaques. Purroy et al. [252] demonstrated that higher levels of MMP-10 correlate with coronary calcifications in subjects with subclinical atherosclerosis. In addition, the investigators observed in Apoe^−/−^Mmp10^−/−^ mice a low rate of atherosclerosis occurrence correlated with reduced systemic and local inflammatory status. 

The role of MMP-10 in vascular calcification in atherosclerosis was also highlighted by Matilla et al. [253]. Calcific aortic valve disease (CAVD) is based on the same pathophysiological changes as in atherosclerosis, lipid infiltration, inflammation, neoangiogenesis, and endothelial dysfunction, representing the central mechanisms underlying the progression of this pathology [254,255]. Jung et al. [256] demonstrated, using MMP-targeted molecular imaging, that MMPs are increased in a murine model of aortic valve disease, but further studies are needed to validate this promising imaging tool to assess the prognosis of these patients. In addition to the proinflammatory role, the active form of MMP-10 also has a fibrotic and osteogenic role, leading to increased expression of TNF-α and IL-1β in valvular interstitial cells derived from human calcified stenotic aortic valves [253].

## 10. Assessing Atherosclerosis through Artificial Intelligence

Artificial intelligence (AI) is a branch of computational science that aims to equal human intellectual processes [257]. From the first concept 60 years ago to the present day, the technology has improved and can now be used in many fields, including medicine [258]. Machine learning (ML) is one the most popular technologies of AI and its role is to find regular patterns behind datasets and to build and test different models in order to predict future data [259]. Medical AI is a growing field with multiple benefits for both the medical industry and healthcare professionals [260]. Recognition of medical images, providing on their basis more reliable imaging diagnostic information and analyzing large datasets that are difficult to analyze by traditional data-processing methods are some of the basic applications of AI [260]. Traditional biostatistics methods provide a limited overview through the information provided by correlations between a single variable and disease. Analysis of biological metrics and analytes sampled from bigger datasets across not only patients but across scales are available through AI, as machine learning can convert raw data into deployable models [261]. In the atherosclerosis research field, ML applications focus on event prediction, risk stratification, diagnostic classification, or biomarker discovery [262,263]. To date, multiple clinical studies have shown applications of ML in image processing associated with atherosclerosis. Data regarding the characterization of plaque components, plaque morphology, or arterial wall measurements were extracted by ML from the analysis of images [264,265]. ML also has practical applications in risk stratification by identifying relevant biomarkers within datasets based on multivariate interacting variables [262]. Munger et al. [266] used ML to determine significant biomarkers for non-calcified coronary burden in patients with psoriasis and concluded that factors such as apolipoprotein A1, HDL-cholesterol, LDL-cholesterol, total cholesterol, apolipoprotein B, and (hs-CRP) correlate positively with obesity, dyslipidemia, and inflammation, thereby contributing to the progression of atherosclerosis. In a similar study, Forné et al. [267] used ML analysis to determine biomarkers for atherosclerosis in patients with chronic kidney disease by using random forest algorithms and concluded that matrix metalloproteinase-9 and vascular endothelial growth factor increase the cardiovascular risk in this population. Ross et al. [268,269] used ML to identify prediction models for assessing the mortality risk and produced a classification with increased clinical applicability in peripheral artery disease. ML can identify changes in gene expression associated with the production of biomarkers associated with atherosclerotic processes, thus contributing to biological diagnostic screening [270]. AI can be applied in genetic screening programs in patients with familial hypercholesterolemia. Correia et al. [271] used AI to identify new biomarkers and develop new models to improve the identification of individuals carrying monogenic causative variants of familial hypercholesterolemia. In their study, the best predicting models included apoB/apoA-I, TG/apoB, and LDL1, biomolecules excluded from classical biomarker panel analysis, and concluded that these parameters are associated with an improved identification of monogenic individuals. AI is a field in continuous development, with multiple applications in cardiovascular diseases, of maximum utility for patient and doctor alike.

## 11. Conclusions—Atherosclerosis as a “Moving Target”

Atherosclerosis is a dynamic, multifactorial process which, through the prism of complex pathophysiological mechanisms, can be seen as a constantly moving target. Identifying it in its early stages, correcting the associated risk factors, and identifying new molecules with both diagnostic and therapeutic roles is a challenge for the scientific community.

## References

[1] Botts S.R., Fish J.E., Howe K.L. (2021). Dysfunctional Vascular Endothelium as a Driver of Atherosclerosis: Emerging Insights into Pathogenesis and Treatment. Front. Pharmacol..

[2] Buliga-Finis O.N., Ouatu A., Badescu M.C., Dima N., Tanase D.M., Richter P., Rezus C. (2022). Beyond the Cardiorenal Syndrome: Pathophysiological Approaches and Biomarkers for Renal and Cardiac Crosstalk. Diagnostics.

[3] Dai H., Much A.A., Maor E., Asher E., Younis A., Xu Y., Lu Y., Liu X., Shu J., Bragazzi N.L. (2022). Global, Regional, and National Burden of Ischaemic Heart Disease and Its Attributable Risk Factors, 1990–2017: Results from the Global Burden of Disease Study 2017. Eur. Heart J.-Qual. Care Clin. Outcomes.

[4] Piepoli M.F., Hoes A.W., Agewall S., Albus C., Brotons C., Catapano A.L., Cooney M.-T., Corrà U., Cosyns B., Deaton C. (2016). 2016 European Guidelines on Cardiovascular Disease Prevention in Clinical Practice: The Sixth Joint Task Force of the European Society of Cardiology and Other Societies on Cardiovascular Disease Prevention in Clinical Practice (Constituted by Representatives of 10 Societies and by Invited Experts) Developed with the Special Contribution of the European Association for Cardiovascular Prevention & Rehabilitation (EACPR). Eur. Heart. J..

[5] Olson R.E. (1998). Discovery of the Lipoproteins, Their Role in Fat Transport and Their Significance as Risk Factors. J. Nutr..

[6] Steinberg D. (2005). Thematic Review Series: The Pathogenesis of Atherosclerosis. An Interpretive History of the Cholesterol Controversy: Part II:The Early Evidence Linking Hypercholesterolemia to Coronary Disease in Humans. J. Lipid Res..

[7] Konstantinov I.E., Jankovic G.M. (2013). Alexander I. Ignatowski: A Pioneer in the Study of Atherosclerosis. Tex. Heart Inst. J..

[8] Seidman M.A., Mitchell R.N., Stone J.R. (2014). Pathophysiology of Atherosclerosis. Cellular and Molecular Pathobiology of Cardiovascular Disease.

[9] Mallika V., Goswami B., Rajappa M. (2007). Atherosclerosis Pathophysiology and the Role of Novel Risk Factors: A Clinicobiochemical Perspective. Angiology.

[10] Cismaru G., Serban T., Tirpe A. (2021). Ultrasound Methods in the Evaluation of Atherosclerosis: From Pathophysiology to Clinic. Biomedicines.

[11] Falk E., Shah P.K., Fuster V. (1995). Coronary Plaque Disruption. Circulation.

[12] Surma S., Czober T., Lepich T., Sierka O., Bajor G. (2020). Selected Biomarkers of Atherosclerosis—Clinical Aspects. Acta Angiol..

[13] Stary H.C., Chandler A.B., Dinsmore R.E., Fuster V., Glagov S., Insull W., Rosenfeld M.E., Schwartz C.J., Wagner W.D., Wissler R.W. (1995). A Definition of Advanced Types of Atherosclerotic Lesions and a Histological Classification of Atherosclerosis: A Report From the Committee on Vascular Lesions of the Council on Arteriosclerosis, American Heart Association. Circulation.

[14] Wang T.J. (2011). Assessing the Role of Circulating, Genetic, and Imaging Biomarkers in Cardiovascular Risk Prediction. Circulation.

[15] Yla-Herttuala S., Bentzon J.F., Daemen M., Falk E., Garcia-Garcia H.M., Herrmann J., Hoefer I., Jauhiainen S., Jukema J.W., Krams R. (2013). Stabilization of Atherosclerotic Plaques: An Update. Eur. Heart J..

[16] Biomarkers Definitions Working Group (2001). Biomarkers and Surrogate Endpoints: Preferred Definitions and Conceptual Framework. Clin. Pharmacol. Ther..

[17] Hoefer I.E., Steffens S., Ala-Korpela M., Bäck M., Badimon L., Bochaton-Piallat M.-L., Boulanger C.M., Caligiuri G., Dimmeler S., Egido J. (2015). Novel Methodologies for Biomarker Discovery in Atherosclerosis. Eur. Heart J..

[18] Wong Y.-K., Tse H.-F. (2021). Circulating Biomarkers for Cardiovascular Disease Risk Prediction in Patients with Cardiovascular Disease. Front. Cardiovasc. Med..

[19] Wielkoszyński T., Zalejska-Fiolka J., Strzelczyk J.K., Owczarek A.J., Cholewka A., Furmański M., Stanek A. (2018). Oxysterols Increase Inflammation, Lipid Marker Levels and Reflect Accelerated Endothelial Dysfunction in Experimental Animals. Mediat. Inflamm..

[20] Wang J., Tan G.-J., Han L.-N., Bai Y.-Y., He M., Liu H.-B. (2017). Novel Biomarkers for Cardiovascular Risk Prediction. J. Geriatr. Cardiol..

[21] Zhang H., Park Y., Wu J., Chen X., Lee S., Yang J., Dellsperger K.C., Zhang C. (2009). Role of TNF-Alpha in Vascular Dysfunction. Clin. Sci..

[22] Mendall M.A., Patel P., Asante M., Ballam L., Morris J., Strachan D.P., Camm A.J., Northfield T.C. (1997). Relation of Serum Cytokine Concentrations to Cardiovascular Risk Factors and Coronary Heart Disease. Heart.

[23] Ridker P.M., Rifai N., Stampfer M.J., Hennekens C.H. (2000). Plasma Concentration of Interleukin-6 and the Risk of Future Myocardial Infarction among Apparently Healthy Men. Circulation.

[24] Reiss A.B., Siegart N.M., de Leon J. (2017). Interleukin-6 in Atherosclerosis: Atherogenic or Atheroprotective?. Clin. Lipidol..

[25] Biscetti F., Nardella E., Cecchini A.L., Flex A., Landolfi R. (2020). Biomarkers of Vascular Disease in Diabetes: The Adipose-Immune System Cross Talk. Intern. Emerg. Med..

[26] Liu S.C., Yi T.C., Weng H.Y., Zhang L., Li Y.X., Mohetaboer M., Zhang Y., Jiang J., Li J.P., Huo Y. (2018). Prognostic value of myeloperoxidase concentration in patients with acute coronary syndrome. Zhonghua Xin Xue Guan Bing Za Zhi.

[27] Ley K., Huo Y. (2001). VCAM-1 Is Critical in Atherosclerosis. J. Clin. Investig..

[28] Varona J.F., Ortiz-Regalón R., Sánchez-Vera I., López-Melgar B., García-Durango C., Castellano Vázquez J.M., Solís J., Fernández-Friera L., Vidal-Vanaclocha F. (2019). Soluble ICAM 1 and VCAM 1 Blood Levels Alert on Subclinical Atherosclerosis in Non Smokers with Asymptomatic Metabolic Syndrome. Arch. Med. Res..

[29] Shrivastava A.K., Singh H.V., Raizada A., Singh S.K. (2015). C-Reactive Protein, Inflammation and Coronary Heart Disease. Egypt. Heart J..

[30] Orsó E., Schmitz G. (2017). Lipoprotein(a) and Its Role in Inflammation, Atherosclerosis and Malignancies. Clin. Res. Cardiol. Suppl..

[31] Stec J.J., Silbershatz H., Tofler G.H., Matheney T.H., Sutherland P., Lipinska I., Massaro J.M., Wilson P.F., Muller J.E., D’Agostino R.B. (2000). Association of Fibrinogen with Cardiovascular Risk Factors and Cardiovascular Disease in the Framingham Offspring Population. Circulation.

[32] Yang R.-L., Shi Y.-H., Hao G., Li W., Le G.-W. (2008). Increasing Oxidative Stress with Progressive Hyperlipidemia in Human: Relation between Malondialdehyde and Atherogenic Index. J. Clin. Biochem. Nutr..

[33] Viigimaa M., Abina J., Zemtsovskaya G., Tikhaze A., Konovalova G., Kumskova E., Lankin V. (2010). Malondialdehyde-Modified Low-Density Lipoproteins as Biomarker for Atherosclerosis. Blood Press..

[34] Alghazeer R., Aboulmeedah E., Elgahmasi S., Alghazir N., Almukthar Z., Enaami M., Rhuma A. (2019). Comparative Evaluation of Antioxidant Enzymes and Serum Selenium Levels in Libyan Atherosclerotic Patients. J. Biosci. Med..

[35] Stanek A., Cholewka A., Wielkoszyński T., Romuk E., Sieroń K., Sieroń A. (2017). Increased Levels of Oxidative Stress Markers, Soluble CD40 Ligand, and Carotid Intima-Media Thickness Reflect Acceleration of Atherosclerosis in Male Patients with Ankylosing Spondylitis in Active Phase and without the Classical Cardiovascular Risk Factors. Oxidative Med. Cell. Longev..

[36] Weckbach L.T., Groesser L., Borgolte J., Pagel J.-I., Pogoda F., Schymeinsky J., Müller-Höcker J., Shakibaei M., Muramatsu T., Deindl E. (2012). Midkine Acts as Proangiogenic Cytokine in Hypoxia-Induced Angiogenesis. Am. J. Physiol.-Heart Circ. Physiol..

[37] Takemoto Y., Horiba M., Harada M., Sakamoto K., Takeshita K., Murohara T., Kadomatsu K., Kamiya K. (2018). Midkine Promotes Atherosclerotic Plaque Formation Through Its Pro-Inflammatory, Angiogenic and Anti-Apoptotic Functions in Apolipoprotein E-Knockout Mice. Circ. J..

[38] Zhang Z.-Z., Wang G., Yin S.-H., Yu X.-H. (2021). Midkine: A Multifaceted Driver of Atherosclerosis. Clin. Chim. Acta.

[39] Guzel S., Cinemre F.B., Guzel E.C., Kucukyalcin V., Kiziler A.R., Cavusoglu C., Gulyasar T., Cinemre H., Aydemir B. (2018). Midkine Levels and Its Relationship with Atherosclerotic Risk Factors in Essential Hypertensive Patients. Niger J. Clin. Pract..

[40] Ou H.-X., Huang Q., Liu C.-H., Xiao J., Lv Y.-C., Li X., Lei L.-P., Mo Z.-C. (2020). Midkine Inhibits Cholesterol Efflux by Decreasing ATP-Binding Membrane Cassette Transport Protein A1 via Adenosine Monophosphate-Activated Protein Kinase/Mammalian Target of Rapamycin Signaling in Macrophages. Circ. J..

[41] Yu X.-H., Fu Y.-C., Zhang D.-W., Yin K., Tang C.-K. (2013). Foam Cells in Atherosclerosis. Clin. Chim. Acta.

[42] Grootaert M.O.J., Moulis M., Roth L., Martinet W., Vindis C., Bennett M.R., De Meyer G.R.Y. (2018). Vascular Smooth Muscle Cell Death, Autophagy and Senescence in Atherosclerosis. Cardiovasc. Res..

[43] Wang J., Uryga A.K., Reinhold J., Figg N., Baker L., Finigan A., Gray K., Kumar S., Clarke M., Bennett M. (2015). Vascular Smooth Muscle Cell Senescence Promotes Atherosclerosis and Features of Plaque Vulnerability. Circulation.

[44] Allahverdian S., Chehroudi A.C., McManus B.M., Abraham T., Francis G.A. (2014). Contribution of Intimal Smooth Muscle Cells to Cholesterol Accumulation and Macrophage-like Cells in Human Atherosclerosis. Circulation.

[45] Basatemur G.L., Jørgensen H.F., Clarke M.C.H., Bennett M.R., Mallat Z. (2019). Vascular Smooth Muscle Cells in Atherosclerosis. Nat. Rev. Cardiol..

[46] Masuda T., Maeda K., Sato W., Kosugi T., Sato Y., Kojima H., Kato N., Ishimoto T., Tsuboi N., Uchimura K. (2017). Growth Factor Midkine Promotes T-Cell Activation through Nuclear Factor of Activated T Cells Signaling and Th1 Cell Differentiation in Lupus Nephritis. Am. J. Pathol..

[47] Kosugi T., Yuzawa Y., Sato W., Arata-Kawai H., Suzuki N., Kato N., Matsuo S., Kadomatsu K. (2007). Midkine Is Involved in Tubulointerstitial Inflammation Associated with Diabetic Nephropathy. Lab. Investig.

[48] Liu J., Thewke D.P., Su Y.R., Linton M.F., Fazio S., Sinensky M.S. (2005). Reduced Macrophage Apoptosis Is Associated with Accelerated Atherosclerosis in Low-Density Lipoprotein Receptor-Null Mice. Arterioscler. Thromb. Vasc. Biol..

[49] Farahi L., Sinha S.K., Lusis A.J. (2021). Roles of Macrophages in Atherogenesis. Front. Pharm..

[50] Erbilgin A., Seldin M.M., Wu X., Mehrabian M., Zhou Z., Qi H., Dabirian K.S., Sevag Packard R.R., Hsieh W., Bensinger S.J. (2018). Transcription Factor Zhx2 Deficiency Reduces Atherosclerosis and Promotes Macrophage Apoptosis in Mice. Arter. Thromb. Vasc. Biol..

[51] Harada M., Hojo M., Kamiya K., Kadomatsu K., Murohara T., Kodama I., Horiba M. (2016). Exogenous Midkine Administration Prevents Cardiac Remodeling in Pacing-Induced Congestive Heart Failure of Rabbits. Heart Vessel..

[52] Kitahara T., Shishido T., Suzuki S., Katoh S., Sasaki T., Ishino M., Nitobe J., Miyamoto T., Miyashita T., Watanabe T. (2010). Serum Midkine as a Predictor of Cardiac Events in Patients with Chronic Heart Failure. J. Card Fail.

[53] Przybylowski P., Malyszko J., Malyszko J.S. (2010). Serum Midkine Is Related to NYHA Class and Cystatin C in Heart Transplant Recipients. Transpl. Proc..

[54] Woulfe K.C., Sucharov C.C. (2017). Midkine’s Role in Cardiac Pathology. J. Cardiovasc. Dev. Dis..

[55] Kadomatsu K., Bencsik P., Görbe A., Csonka C., Sakamoto K., Kishida S., Ferdinandy P. (2014). Therapeutic Potential of Midkine in Cardiovascular Disease. Br. J. Pharm..

[56] Becker R.C., Owens A.P., Sadayappan S. (2020). Tissue-Level Inflammation and Ventricular Remodeling in Hypertrophic Cardiomyopathy. J. Thromb. Thrombolysis.

[57] Salaru D.L., Albert C., Königsmark U., Brandt S., Halloul Z., Heller A., Heidecke H., Dragun D., Mertens P.R. (2014). Serum Levels for Midkine, a Heparin-Binding Growth Factor, Inversely Correlate with Angiotensin and Endothelin Receptor Autoantibody Titers in Patients with Macroangiopathy. Int. Angiol..

[58] Chen Z., Zhu Y., Zhang L. (2020). Study of Three Novel Biomarkers, MR-ProADM, Midkine, and Stromelysin2, and Peripheral Atherosclerosis in a Chinese Han Population: A Case-Control Study. Eur. J. Inflamm..

[59] Smolarz B., Durczyński A., Romanowicz H., Szyłło K., Hogendorf P. (2022). MiRNAs in Cancer (Review of Literature). Int. J. Mol. Sci..

[60] Lee L.W., Zhang S., Etheridge A., Ma L., Martin D., Galas D., Wang K. (2010). Complexity of the MicroRNA Repertoire Revealed by Next-Generation Sequencing. RNA.

[61] Zhang R., Su B. (2009). Small but Influential: The Role of MicroRNAs on Gene Regulatory Network and 3′UTR Evolution. J. Genet. Genom..

[62] Rossbach M. (2010). Small Non-Coding RNAs as Novel Therapeutics. Curr. Mol. Med..

[63] Pozniak T., Shcharbin D., Bryszewska M. (2022). Circulating MicroRNAs in Medicine. Int. J. Mol. Sci..

[64] Gilad S., Meiri E., Yogev Y., Benjamin S., Lebanony D., Yerushalmi N., Benjamin H., Kushnir M., Cholakh H., Melamed N. (2008). Serum MicroRNAs Are Promising Novel Biomarkers. PLoS ONE.

[65] Weber J.A., Baxter D.H., Zhang S., Huang D.Y., Huang K.H., Lee M.J., Galas D.J., Wang K. (2010). The MicroRNA Spectrum in 12 Body Fluids. Clin. Chem..

[66] Solly E.L., Dimasi C.G., Bursill C.A., Psaltis P.J., Tan J.T.M. (2019). MicroRNAs as Therapeutic Targets and Clinical Biomarkers in Atherosclerosis. JCM.

[67] Vickers K.C., Palmisano B.T., Shoucri B.M., Shamburek R.D., Remaley A.T. (2011). MicroRNAs Are Transported in Plasma and Delivered to Recipient Cells by High-Density Lipoproteins. Nat. Cell Biol..

[68] Glass C.K., Witztum J.L. (2001). Atherosclerosis: The Road Ahead. Cell.

[69] Pentikäinen M.O., Öörni K., Ala-Korpela M., Kovanen P.T. (2000). Modified LDL – Trigger of Atherosclerosis and Inflammation in the Arterial Intima. J. Intern. Med..

[70] Esau C., Davis S., Murray S.F., Yu X.X., Pandey S.K., Pear M., Watts L., Booten S.L., Graham M., McKay R. (2006). MiR-122 Regulation of Lipid Metabolism Revealed by in Vivo Antisense Targeting. Cell Metab..

[71] Sun X., Belkin N., Feinberg M.W. (2013). Endothelial MicroRNAs and Atherosclerosis. Curr. Atheroscler. Rep..

[72] Sun X., He S., Wara A.K.M., Icli B., Shvartz E., Tesmenitsky Y., Belkin N., Li D., Blackwell T.S., Sukhova G.K. (2014). Systemic Delivery of MicroRNA-181b Inhibits Nuclear Factor-ΚB Activation, Vascular Inflammation, and Atherosclerosis in Apolipoprotein E–Deficient Mice. Circ. Res..

[73] Su Y., Yuan J., Zhang F., Lei Q., Zhang T., Li K., Guo J., Hong Y., Bu G., Lv X. (2019). MicroRNA-181a-5p and MicroRNA-181a-3p Cooperatively Restrict Vascular Inflammation and Atherosclerosis. Cell Death Dis..

[74] Shu Z., Tan J., Miao Y., Zhang Q. (2019). The Role of Microvesicles Containing MicroRNAs in Vascular Endothelial Dysfunction. J. Cell Mol. Med..

[75] György B., Szabó T.G., Pásztói M., Pál Z., Misják P., Aradi B., László V., Pállinger E., Pap E., Kittel A. (2011). Membrane Vesicles, Current State-of-the-Art: Emerging Role of Extracellular Vesicles. Cell Mol. Life Sci..

[76] Kosaka N., Iguchi H., Hagiwara K., Yoshioka Y., Takeshita F., Ochiya T. (2013). Neutral Sphingomyelinase 2 (NSMase2)-Dependent Exosomal Transfer of Angiogenic MicroRNAs Regulate Cancer Cell Metastasis. J. Biol. Chem..

[77] Kosaka N., Iguchi H., Yoshioka Y., Takeshita F., Matsuki Y., Ochiya T. (2010). Secretory Mechanisms and Intercellular Transfer of MicroRNAs in Living Cells. J. Biol. Chem..

[78] Jansen F., Yang X., Proebsting S., Hoelscher M., Przybilla D., Baumann K., Schmitz T., Dolf A., Endl E., Franklin B.S. (2014). MicroRNA Expression in Circulating Microvesicles Predicts Cardiovascular Events in Patients with Coronary Artery Disease. J. Am. Heart Assoc..

[79] Peluso I., Morabito G., Urban L., Ioannone F., Serafini M. (2012). Oxidative Stress in Atherosclerosis Development: The Central Role of LDL and Oxidative Burst. Endocr. Metab. Immune Disord. Drug Targets.

[80] Förstermann U., Xia N., Li H. (2017). Roles of Vascular Oxidative Stress and Nitric Oxide in the Pathogenesis of Atherosclerosis. Circ. Res..

[81] Joris V., Gomez E.L., Menchi L., Lobysheva I., Di Mauro V., Esfahani H., Condorelli G., Balligand J.-L., Catalucci D., Dessy C. (2018). MicroRNA-199a-3p and MicroRNA-199a-5p Take Part to a Redundant Network of Regulation of the NOS (NO Synthase)/NO Pathway in the Endothelium. Arterioscler. Thromb. Vasc. Biol..

[82] Hsu C.-Y., Chen Y.-T., Su Y.-W., Chang C.-C., Huang P.-H., Lin S.-J. (2017). Statin Therapy Reduces Future Risk of Lower-Limb Amputation in Patients with Diabetes and Peripheral Artery Disease. J. Clin. Endocrinol. Metab..

[83] Yang S., Mi X., Chen Y., Feng C., Hou Z., Hui R., Zhang W. (2018). MicroRNA-216a Induces Endothelial Senescence and Inflammation via Smad3/IκBα Pathway. J. Cell. Mol. Med..

[84] Nguyen M.-A., Karunakaran D., Geoffrion M., Cheng H.S., Tandoc K., Perisic Matic L., Hedin U., Maegdefessel L., Fish J.E., Rayner K.J. (2018). Extracellular Vesicles Secreted by Atherogenic Macrophages Transfer MicroRNA to Inhibit Cell Migration. Arter. Thromb. Vasc. Biol..

[85] Afonyushkin T., Binder C.J. (2018). Extracellular Vesicles Act as Messengers of Macrophages Sensing Atherogenic Stimuli. Arter. Thromb. Vasc. Biol..

[86] Wang D., Atanasov A.G. (2019). The MicroRNAs Regulating Vascular Smooth Muscle Cell Proliferation: A Minireview. Int. J. Mol. Sci..

[87] Alshanwani A.R., Riches-Suman K., O’Regan D.J., Wood I.C., Turner N.A., Porter K.E. (2018). MicroRNA-21 Drives the Switch to a Synthetic Phenotype in Human Saphenous Vein Smooth Muscle Cells. IUBMB Life.

[88] MicroRNA-1 Inhibits Myocardin-Induced Contractility of Human Vascular Smooth Muscle Cells-Jiang-2010-Journal of Cellular Physiology-Wiley Online Library. https://onlinelibrary.wiley.com/doi/10.1002/jcp.22230.

[89] Lin Y., Liu X., Cheng Y., Yang J., Huo Y., Zhang C. (2009). Involvement of MicroRNAs in Hydrogen Peroxide-Mediated Gene Regulation and Cellular Injury Response in Vascular Smooth Muscle Cells *. J. Biol. Chem..

[90] Jin H., Li D.Y., Chernogubova E., Sun C., Busch A., Eken S.M., Saliba-Gustafsson P., Winter H., Winski G., Raaz U. (2018). Local Delivery of MiR-21 Stabilizes Fibrous Caps in Vulnerable Atherosclerotic Lesions. Mol. Ther..

[91] Eken S.M., Jin H., Chernogubova E., Li Y., Simon N., Sun C., Korzunowicz G., Busch A., Bäcklund A., Österholm C. (2017). MicroRNA-210 Enhances Fibrous Cap Stability in Advanced Atherosclerotic Lesions. Circ. Res..

[92] Sedding D.G., Boyle E.C., Demandt J.A.F., Sluimer J.C., Dutzmann J., Haverich A., Bauersachs J. (2018). Vasa Vasorum Angiogenesis: Key Player in the Initiation and Progression of Atherosclerosis and Potential Target for the Treatment of Cardiovascular Disease. Front. Immunol..

[93] Boyle E.C., Sedding D.G., Haverich A. (2017). Targeting Vasa Vasorum Dysfunction to Prevent Atherosclerosis. Vasc. Pharm..

[94] Urbich C., Kuehbacher A., Dimmeler S. (2008). Role of MicroRNAs in Vascular Diseases, Inflammation, and Angiogenesis. Cardiovasc. Res..

[95] Wu M., Xun M., Chen Y. (2022). Circular RNAs: Regulators of Vascular Smooth Muscle Cells in Cardiovascular Diseases. J. Mol. Med..

[96] Parahuleva M.S., Lipps C., Parviz B., Hölschermann H., Schieffer B., Schulz R., Euler G. (2018). MicroRNA Expression Profile of Human Advanced Coronary Atherosclerotic Plaques. Sci. Rep..

[97] Pereira-Silva D., Carneiro F., Almeida K., Fernandes-Santos C. (2018). Role of MiRNAs on the Pathophysiology of Cardiovascular Diseases. Arq. Bras. Cardiol..

[98] Zhang X., Shao S., Geng H., Yu Y., Wang C., Liu Z., Yu C., Jiang X., Deng Y., Gao L. (2014). Expression Profiles of Six Circulating MicroRNAs Critical to Atherosclerosis in Patients with Subclinical Hypothyroidism: A Clinical Study. J. Clin. Endocrinol. Metab..

[99] Pereira-da-Silva T., Coutinho Cruz M., Carrusca C., Cruz Ferreira R., Napoleão P., Mota Carmo M. (2018). Circulating MicroRNA Profiles in Different Arterial Territories of Stable Atherosclerotic Disease: A Systematic Review. Am. J. Cardiovasc. Dis..

[100] Fichtlscherer S., De Rosa S., Fox H., Schwietz T., Fischer A., Liebetrau C., Weber M., Hamm C.W., Röxe T., Müller-Ardogan M. (2010). Circulating MicroRNAs in Patients with Coronary Artery Disease. Circ. Res..

[101] Wang X., Lian Y., Wen X., Guo J., Wang Z., Jiang S., Hu Y. (2017). Expression of MiR-126 and Its Potential Function in Coronary Artery Disease. Afr. Health Sci..

[102] Weber M., Baker M.B., Patel R.S., Quyyumi A.A., Bao G., Searles C.D. (2011). MicroRNA Expression Profile in CAD Patients and the Impact of ACEI/ARB. Cardiol. Res. Pract..

[103] Zhao J., Wang Q., Zhu R., Yang J. (2022). Circulating Non-Coding RNAs as Potential Biomarkers for Ischemic Stroke: A Systematic Review. J. Mol. Neurosci..

[104] Akella A., Bhattarai S., Dharap A. (2019). Long Noncoding RNAs in the Pathophysiology of Ischemic Stroke. Neuromolecular Med..

[105] Chen J., Liu P., Dong X., Jin J., Xu Y. (2021). The Role of LncRNAs in Ischemic Stroke. Neurochem. Int..

[106] Yang S., Zhan X., He M., Wang J., Qiu X. (2020). MiR-135b Levels in the Peripheral Blood Serve as a Marker Associated with Acute Ischemic Stroke. Exp. Med..

[107] Cheng X., Kan P., Ma Z., Wang Y., Song W., Huang C., Zhang B. (2018). Exploring the Potential Value of MiR-148b-3p, MiR-151b and MiR-27b-3p as Biomarkers in Acute Ischemic Stroke. Biosci. Rep..

[108] Mahjoob G., Ahmadi Y., Fatima Rajani H., Khanbabaei N., Abolhasani S. (2022). Circulating MicroRNAs as Predictive Biomarkers of Coronary Artery Diseases in Type 2 Diabetes Patients. J. Clin. Lab. Anal..

[109] Du Y., Yang S.H., Li S., Cui C.J., Zhang Y., Zhu C.G., Guo Y.L., Wu N.Q., Gao Y., Sun J. (2016). Circulating MicroRNAs as Novel Diagnostic Biomarkers for Very Early-Onset (≤40 Years) Coronary Artery Disease. Biomed. Environ. Sci..

[110] Zhang P., Liang T., Chen Y., Wang X., Wu T., Xie Z., Luo J., Yu Y., Yu H. (2020). Circulating Exosomal MiRNAs as Novel Biomarkers for Stable Coronary Artery Disease. Biomed. Res. Int..

[111] Šatrauskienė A., Navickas R., Laucevičius A., Krilavičius T., Užupytė R., Zdanytė M., Ryliškytė L., Jucevičienė A., Holvoet P. (2021). Mir-1, MiR-122, MiR-132, and MiR-133 Are Related to Subclinical Aortic Atherosclerosis Associated with Metabolic Syndrome. Int. J. Environ. Res. Public Health.

[112] Xu K., Chen C., Wu Y., Wu M., Lin L. (2021). Advances in MiR-132-Based Biomarker and Therapeutic Potential in the Cardiovascular System. Front. Pharm..

[113] Li H., Zhang P., Li F., Yuan G., Wang X., Zhang A., Li F. (2019). Plasma MiR-22-5p, MiR-132-5p, and MiR-150-3p Are Associated with Acute Myocardial Infarction. BioMed Res. Int..

[114] Singh S., de Ronde M.W.J., Kok M.G.M., Beijk M.A., De Winter R.J., van der Wal A.C., Sondermeijer B.M., Meijers J.C.M., Creemers E.E., Pinto-Sietsma S.-J. (2020). MiR-223-3p and MiR-122-5p as Circulating Biomarkers for Plaque Instability. Open Heart.

[115] Hromadka M., Motovska Z., Hlinomaz O., Kala P., Tousek F., Jarkovsky J., Beranova M., Jansky P., Svoboda M., Krepelkova I. (2021). MiR-126-3p and MiR-223-3p as Biomarkers for Prediction of Thrombotic Risk in Patients with Acute Myocardial Infarction and Primary Angioplasty. J. Pers. Med..

[116] Jebari-Benslaiman S., Galicia-García U., Larrea-Sebal A., Olaetxea J.R., Alloza I., Vandenbroeck K., Benito-Vicente A., Martín C. (2022). Pathophysiology of Atherosclerosis. Int. J. Mol. Sci..

[117] Zeng Z., Zhu Q., Zhao Z., Zu X., Liu J. (2021). Magic and Mystery of MicroRNA-32. J. Cell Mol. Med..

[118] Zhang X., Cai H., Zhu M., Qian Y., Lin S., Li X. (2020). Circulating MicroRNAs as Biomarkers for Severe Coronary Artery Disease. Medicine.

[119] Rozhkova A.V., Dmitrieva V.G., Nosova E.V., Dergunov A.D., Limborska S.A., Dergunova L.V. (2021). Genomic Variants and Multilevel Regulation of ABCA1, ABCG1, and SCARB1 Expression in Atherogenesis. J. Cardiovasc. Dev. Dis..

[120] Schumacher D., Curaj A., Simsekyilmaz S., Schober A., Liehn E.A., Mause S.F. (2021). MiR155 Deficiency Reduces Myofibroblast Density but Fails to Improve Cardiac Function after Myocardial Infarction in Dyslipidemic Mouse Model. Int. J. Mol. Sci..

[121] Li J., Cai S.X., He Q., Zhang H., Friedberg D., Wang F., Redington A.N. (2018). Intravenous MiR-144 Reduces Left Ventricular Remodeling after Myocardial Infarction. Basic Res. Cardiol..

[122] Casula M., Montecucco F., Bonaventura A., Liberale L., Vecchié A., Dallegri F., Carbone F. (2017). Update on the Role of Pentraxin 3 in Atherosclerosis and Cardiovascular Diseases. Vasc. Pharmacol..

[123] Pepys M.B., Baltz M.L., Dixon F.J., Kunkel H.G. (1983). Acute Phase Proteins with Special Reference to C-Reactive Protein and Related Proteins (Pentaxins) and Serum Amyloid A Protein. Advances in Immunology.

[124] Richter P., Burlui A., Bratoiu I., Cardoneanu A., Rezus C., Rezus E. (2021). A Review of Anti-C Reactive Protein Antibodies in Systemic Lupus Erythematosus. J. Interdiscip. Med..

[125] Vilahur G., Badimon L. (2015). Biological Actions of Pentraxins. Vasc. Pharmacol..

[126] Kunes P., Holubcova Z., Kolackova M., Krejsek J. (2012). Pentraxin 3(PTX 3): An Endogenous Modulator of the Inflammatory Response. Mediat. Inflamm..

[127] Deban L., Russo R.C., Sironi M., Moalli F., Scanziani M., Zambelli V., Cuccovillo I., Bastone A., Gobbi M., Valentino S. (2010). Regulation of Leukocyte Recruitment by the Long Pentraxin PTX3. Nat. Immunol..

[128] de la Torre Y.M., Fabbri M., Jaillon S., Bastone A., Nebuloni M., Vecchi A., Mantovani A., Garlanda C. (2010). Evolution of the Pentraxin Family: The New Entry PTX4. J. Immunol..

[129] Klouche M., Peri G., Knabbe C., Eckstein H.-H., Schmid F.-X., Schmitz G., Mantovani A. (2004). Modified Atherogenic Lipoproteins Induce Expression of Pentraxin-3 by Human Vascular Smooth Muscle Cells. Atherosclerosis.

[130] Napoleone E., Di Santo A., Bastone A., Peri G., Mantovani A., de Gaetano G., Donati M.B., Lorenzet R. (2002). Long Pentraxin PTX3 Upregulates Tissue Factor Expression in Human Endothelial Cells: A Novel Link between Vascular Inflammation and Clotting Activation. Arter. Thromb. Vasc. Biol..

[131] Napoleone E., di Santo A., Peri G., Mantovani A., de Gaetano G., Donati M.B., Lorenzet R. (2004). The Long Pentraxin PTX3 Up-Regulates Tissue Factor in Activated Monocytes: Another Link between Inflammation and Clotting Activation. J. Leukoc. Biol..

[132] Zlibut A., Bocsan I.C., Agoston-Coldea L. (2019). Pentraxin-3 and Endothelial Dysfunction. Advances in Clinical Chemistry.

[133] O’Neill C.L., Guduric-Fuchs J., Chambers S.E.J., O’Doherty M., Bottazzi B., Stitt A.W., Medina R.J. (2016). Endothelial Cell-Derived Pentraxin 3 Limits the Vasoreparative Therapeutic Potential of Circulating Angiogenic Cells. Cardiovasc. Res..

[134] Jenny N.S., Arnold A.M., Kuller L.H., Tracy R.P., Psaty B.M. (2009). Associations of Pentraxin 3 with Cardiovascular Disease and All-Cause Death: The Cardiovascular Health Study. Arter. Thromb. Vasc. Biol..

[135] Jenny N.S., Blumenthal R.S., Kronmal R.A., Rotter J.I., Siscovick D.S., Psaty B.M. (2014). Associations of Pentraxin 3 with Cardiovascular Disease: The Multi-Ethnic Study of Atherosclerosis. J. Thromb. Haemost..

[136] Ogawa T., Kawano Y., Imamura T., Kawakita K., Sagara M., Matsuo T., Kakitsubata Y., Ishikawa T., Kitamura K., Hatakeyama K. (2010). Reciprocal Contribution of Pentraxin 3 and C-Reactive Protein to Obesity and Metabolic Syndrome. Obesity.

[137] Inoue K., Sugiyama A., Reid P.C., Ito Y., Miyauchi K., Mukai S., Sagara M., Miyamoto K., Satoh H., Kohno I. (2007). Establishment of a High Sensitivity Plasma Assay for Human Pentraxin3 as a Marker for Unstable Angina Pectoris. Arter. Thromb. Vasc. Biol..

[138] Kotooka N., Inoue T., Aoki S., Anan M., Komoda H., Node K. (2008). Prognostic Value of Pentraxin 3 in Patients with Chronic Heart Failure. Int. J. Cardiol..

[139] Savchenko A., Imamura M., Ohashi R., Jiang S., Kawasaki T., Hasegawa G., Emura I., Iwanari H., Sagara M., Tanaka T. (2008). Expression of Pentraxin 3 (PTX3) in Human Atherosclerotic Lesions. J. Pathol..

[140] Nerkiz P., Doganer Y.C., Aydogan U., Akbulut H., Parlak A., Aydogdu A., Sari O., Cayci T., Barcin C., Koc B. (2015). Serum Pentraxin-3 Level in Patients Who Underwent Coronary Angiography and Relationship with Coronary Atherosclerosis. Med. Princ. Pract..

[141] Ristagno G., Fumagalli F., Bottazzi B., Mantovani A., Olivari D., Novelli D., Latini R. (2019). Pentraxin 3 in Cardiovascular Disease. Front. Immunol..

[142] Salio M., Chimenti S., De Angelis N., Molla F., Maina V., Nebuloni M., Pasqualini F., Latini R., Garlanda C., Mantovani A. (2008). Cardioprotective Function of the Long Pentraxin PTX3 in Acute Myocardial Infarction. Circulation.

[143] Norata G.D., Garlanda C., Catapano A.L. (2010). The Long Pentraxin PTX3: A Modulator of the Immunoinflammatory Response in Atherosclerosis and Cardiovascular Diseases. Trends Cardiovasc. Med..

[144] Nauta A.J., Bottazzi B., Mantovani A., Salvatori G., Kishore U., Schwaeble W.J., Gingras A.R., Tzima S., Vivanco F., Egido J. (2003). Biochemical and Functional Characterization of the Interaction between Pentraxin 3 and C1q. Eur. J. Immunol..

[145] Yasunaga T., Ikeda S., Koga S., Nakata T., Yoshida T., Masuda N., Kohno S., Maemura K. (2014). Plasma Pentraxin 3 Is a More Potent Predictor of Endothelial Dysfunction than High-Sensitive C-Reactive Protein. Int. Heart J..

[146] Carrizzo A., Lenzi P., Procaccini C., Damato A., Biagioni F., Ambrosio M., Amodio G., Remondelli P., Del Giudice C., Izzo R. (2015). Pentraxin 3 Induces Vascular Endothelial Dysfunction Through a P-Selectin/Matrix Metalloproteinase-1 Pathway. Circulation.

[147] Latini R., Maggioni A.P., Peri G., Gonzini L., Lucci D., Mocarelli P., Vago L., Pasqualini F., Signorini S., Soldateschi D. (2004). Prognostic Significance of the Long Pentraxin PTX3 in Acute Myocardial Infarction. Circulation.

[148] Agrotis A., Kanellakis P., Kostolias G., Di Vitto G., Wei C., Hannan R., Jennings G., Bobik A. (2004). Proliferation of Neointimal Smooth Muscle Cells after Arterial Injury: Dependence on Interactions Between Fibroblast Growth Factor Receptor-2 and Fibroblast Growth Factor-9. J. Biol. Chem..

[149] Jackson C.L., Reidy M.A. (1993). Basic Fibroblast Growth Factor: Its Role in the Control of Smooth Muscle Cell Migration. Am. J. Pathol..

[150] Fornai F., Carrizzo A., Forte M., Ambrosio M., Damato A., Ferrucci M., Biagioni F., Busceti C., Puca A.A., Vecchione C. (2016). The Inflammatory Protein Pentraxin 3 in Cardiovascular Disease. Immun. Ageing.

[151] Mutlu M., Yuksel N., Takmaz T., Dincel A.S., Bilgihan A., Altınkaynak H. (2017). Aqueous Humor Pentraxin-3 Levels in Patients with Diabetes Mellitus. Eye.

[152] Akgul O., Baycan O.F., Bulut U., Somuncu M.U., Pusuroglu H., Ozyilmaz S., Gul M., Demir A.R., Yılmaz E., Yazan S. (2015). Long-Term Prognostic Value of Elevated Pentraxin 3 in Patients Undergoing Primary Angioplasty for ST-Elevation Myocardial Infarction. Coron. Artery Dis..

[153] Kimura S., Inagaki H., Haraguchi G., Sugiyama T., Miyazaki T., Hatano Y., Yoshikawa S., Ashikaga T., Isobe M. (2014). Relationships of Elevated Systemic Pentraxin-3 Levels with High-Risk Coronary Plaque Components and Impaired Myocardial Perfusion After Percutaneous Coronary Intervention in Patients With ST-Elevation Acute Myocardial Infarction. Circ. J..

[154] Guo R., Li Y., Wen J., Li W., Xu Y. (2014). Elevated Plasma Level of Pentraxin-3 Predicts In-Hospital and 30-Day Clinical Outcomes in Patients with Non-ST-Segment Elevation Myocardial Infarction Who Have Undergone Percutaneous Coronary Intervention. CRD.

[155] Matsubara J., Sugiyama S., Nozaki T., Sugamura K., Konishi M., Ohba K., Matsuzawa Y., Akiyama E., Yamamoto E., Sakamoto K. (2011). Pentraxin 3 Is a New Inflammatory Marker Correlated with Left Ventricular Diastolic Dysfunction and Heart Failure with Normal Ejection Fraction. J. Am. Coll. Cardiol..

[156] Dubin R., Li Y., Ix J.H., Shlipak M.G., Whooley M., Peralta C.A. (2012). Associations of Pentraxin-3 with Cardiovascular Events, Incident Heart Failure, and Mortality among Persons with Coronary Heart Disease: Data from the Heart and Soul Study. Am. Heart J..

[157] Charla E., Mercer J., Maffia P., Nicklin S.A. (2020). Extracellular Vesicle Signalling in Atherosclerosis. Cell Signal.

[158] Yates A.G., Pink R.C., Erdbrügger U., Siljander P.R.-M., Dellar E.R., Pantazi P., Akbar N., Cooke W.R., Vatish M., Dias-Neto E. (2022). In Sickness and in Health: The Functional Role of Extracellular Vesicles in Physiology and Pathology in Vivo: Part II: Pathology: Part II: Pathology. J. Extracell Vesicles.

[159] Dorobantu M., Simionescu M., Popa-Fotea N.-M. (2021). Molecular Research in Cardiovascular Disease. Int. J. Mol. Sci..

[160] Coly P.-M., Boulanger C.M. (2022). Role of Extracellular Vesicles in Atherosclerosis: An Update. J. Leukoc. Biol..

[161] Dignat-George F., Boulanger C.M. (2011). The Many Faces of Endothelial Microparticles. ATVB.

[162] Deng W., Tang T., Hou Y., Zeng Q., Wang Y., Fan W., Qu S. (2019). Extracellular Vesicles in Atherosclerosis. Clin. Chim. Acta.

[163] Xu H., Ni Y.-Q., Liu Y.-S. (2021). Mechanisms of Action of MiRNAs and LncRNAs in Extracellular Vesicle in Atherosclerosis. Front Cardiovasc. Med..

[164] Bobryshev Y.V., Killingsworth M.C., Orekhov A.N. (2013). Increased Shedding of Microvesicles from Intimal Smooth Muscle Cells in Athero-Prone Areas of the Human Aorta: Implications for Understanding of the Predisease Stage. Pathobiology.

[165] Perrotta I., Aquila S. (2016). Exosomes in Human Atherosclerosis: An Ultrastructural Analysis Study. Ultrastruct. Pathol..

[166] Arteaga R.B., Chirinos J.A., Soriano A.O., Jy W., Horstman L., Jimenez J.J., Mendez A., Ferreira A., de Marchena E., Ahn Y.S. (2006). Endothelial Microparticles and Platelet and Leukocyte Activation in Patients with the Metabolic Syndrome. Am. J. Cardiol..

[167] Amabile N., Cheng S., Renard J.M., Larson M.G., Ghorbani A., McCabe E., Griffin G., Guerin C., Ho J.E., Shaw S.Y. (2014). Association of Circulating Endothelial Microparticles with Cardiometabolic Risk Factors in the Framingham Heart Study. Eur. Heart J..

[168] Li C.-J., Liu Y., Chen Y., Yu D., Williams K.J., Liu M.-L. (2013). Novel Proteolytic Microvesicles Released from Human Macrophages after Exposure to Tobacco Smoke. Am. J. Pathol..

[169] Ferreira A.C., Peter A.A., Mendez A.J., Jimenez J.J., Mauro L.M., Chirinos J.A., Ghany R., Virani S., Garcia S., Horstman L.L. (2004). Postprandial Hypertriglyceridemia Increases Circulating Levels of Endothelial Cell Microparticles. Circulation.

[170] Nomura S., Shouzu A., Omoto S., Nishikawa M., Iwasaka T. (2004). Effects of Losartan and Simvastatin on Monocyte-Derived Microparticles in Hypertensive Patients with and without Type 2 Diabetes Mellitus. Clin. Appl. Thromb. Hemost..

[171] Konkoth A., Saraswat R., Dubrou C., Sabatier F., Leroyer A.S., Lacroix R., Duchez A.-C., Dignat-George F. (2021). Multifaceted Role of Extracellular Vesicles in Atherosclerosis. Atherosclerosis.

[172] de Freitas R.C.C., Hirata R.D.C., Hirata M.H., Aikawa E. (2021). Circulating Extracellular Vesicles as Biomarkers and Drug Delivery Vehicles in Cardiovascular Diseases. Biomolecules.

[173] Nozaki T., Sugiyama S., Koga H., Sugamura K., Ohba K., Matsuzawa Y., Sumida H., Matsui K., Jinnouchi H., Ogawa H. (2009). Significance of a Multiple Biomarkers Strategy Including Endothelial Dysfunction to Improve Risk Stratification for Cardiovascular Events in Patients at High Risk for Coronary Heart Disease. J. Am. Coll. Cardiol..

[174] Lässer C., Seyed Alikhani V., Ekström K., Eldh M., Torregrosa Paredes P., Bossios A., Sjöstrand M., Gabrielsson S., Lötvall J., Valadi H. (2011). Human Saliva, Plasma and Breast Milk Exosomes Contain RNA: Uptake by Macrophages. J. Transl. Med..

[175] Bernal-Mizrachi L., Jy W., Jimenez J.J., Pastor J., Mauro L.M., Horstman L.L., de Marchena E., Ahn Y.S. (2003). High Levels of Circulating Endothelial Microparticles in Patients with Acute Coronary Syndromes. Am. Heart J..

[176] Leroyer A.S., Isobe H., Lesèche G., Castier Y., Wassef M., Mallat Z., Binder B.R., Tedgui A., Boulanger C.M. (2007). Cellular Origins and Thrombogenic Activity of Microparticles Isolated from Human Atherosclerotic Plaques. J. Am. Coll. Cardiol..

[177] Berezin A.E., Berezin A.A. (2022). Extracellular Vesicles and Thrombogenicity in Atrial Fibrillation. Int. J. Mol. Sci..

[178] Tang N., Bai H., Chen X., Gong J., Li D., Sun Z. (2020). Anticoagulant Treatment Is Associated with Decreased Mortality in Severe Coronavirus Disease 2019 Patients with Coagulopathy. J. Thromb. Haemost..

[179] Aharon A., Tamari T., Brenner B. (2008). Monocyte-Derived Microparticles and Exosomes Induce Procoagulant and Apoptotic Effects on Endothelial Cells. Thromb. Haemost..

[180] Wang J.-G., Williams J.C., Davis B.K., Jacobson K., Doerschuk C.M., Ting J.P.-Y., Mackman N. (2011). Monocytic Microparticles Activate Endothelial Cells in an IL-1β–Dependent Manner. Blood.

[181] Saheera S., Jani V.P., Witwer K.W., Kutty S. (2021). Extracellular Vesicle Interplay in Cardiovascular Pathophysiology. Am. J. Physiol. Heart Circ. Physiol..

[182] Buffolo F., Monticone S., Camussi G., Aikawa E. (2022). Role of Extracellular Vesicles in the Pathogenesis of Vascular Damage. Hypertension.

[183] Ramírez R., Ceprian N., Figuer A., Valera G., Bodega G., Alique M., Carracedo J. (2022). Endothelial Senescence and the Chronic Vascular Diseases: Challenges and Therapeutic Opportunities in Atherosclerosis. J. Pers. Med..

[184] Sun X., Feinberg M.W. (2021). Vascular Endothelial Senescence: Pathobiological Insights, Emerging Long Noncoding RNA Targets, Challenges and Therapeutic Opportunities. Front. Physiol..

[185] Lenasi H. (2018). Endothelial Dysfunction: Old Concepts and New Challenges.

[186] Wu C.-M., Zheng L., Wang Q., Hu Y.-W. (2021). The Emerging Role of Cell Senescence in Atherosclerosis. Clin. Chem. Lab. Med. (CCLM).

[187] Buendía P., Montes de Oca A., Madueño J.A., Merino A., Martín-Malo A., Aljama P., Ramírez R., Rodríguez M., Carracedo J. (2015). Endothelial Microparticles Mediate Inflammation-Induced Vascular Calcification. FASEB J..

[188] Bennett M.R., Sinha S., Owens G.K. (2016). Vascular Smooth Muscle Cells in Atherosclerosis. Circ. Res..

[189] Goetzl E.J., Schwartz J.B., Mustapic M., Lobach I.V., Daneman R., Abner E.L., Jicha G.A. (2017). Altered Cargo Proteins of Human Plasma Endothelial Cell–Derived Exosomes in Atherosclerotic Cerebrovascular Disease. FASEB J..

[190] Chen A., Wang H., Su Y., Zhang C., Qiu Y., Zhou Y., Wan Y., Hu B., Li Y. (2021). Exosomes: Biomarkers and Therapeutic Targets of Diabetic Vascular Complications. Front. Endocrinol..

[191] Chen J., Zhang Q., Liu D., Liu Z. (2021). Exosomes: Advances, Development and Potential Therapeutic Strategies in Diabetic Nephropathy. Metabolism.

[192] Zhang H., Liu J., Qu D., Wang L., Wong C.M., Lau C.-W., Huang Y., Wang Y.F., Huang H., Xia Y. (2018). Serum Exosomes Mediate Delivery of Arginase 1 as a Novel Mechanism for Endothelial Dysfunction in Diabetes. Proc. Natl. Acad. Sci. USA.

[193] Hu W., Song X., Yu H., Sun J., Zhao Y. (2020). Therapeutic Potentials of Extracellular Vesicles for the Treatment of Diabetes and Diabetic Complications. Int. J. Mol. Sci..

[194] Heo J., Kang H. (2022). Exosome-Based Treatment for Atherosclerosis. Int. J. Mol. Sci..

[195] Libby P., Tabas I., Fredman G., Fisher E.A. (2014). Inflammation and Its Resolution as Determinants of Acute Coronary Syndromes. Circ. Res..

[196] Schroder K., Tschopp J. (2010). The Inflammasomes. Cell.

[197] Duewell P., Kono H., Rayner K.J., Sirois C.M., Vladimer G., Bauernfeind F.G., Abela G.S., Franchi L., Nuñez G., Schnurr M. (2010). NLRP3 Inflammasomes Are Required for Atherogenesis and Activated by Cholesterol Crystals. Nature.

[198] Altaf A., Qu P., Zhao Y., Wang H., Lou D., Niu N. (2015). NLRP3 Inflammasome in Peripheral Blood Monocytes of Acute Coronary Syndrome Patients and Its Relationship with Statins. Coron. Artery Dis..

[199] Shi X., Xie W.-L., Kong W.-W., Chen D., Qu P. (2015). Expression of the NLRP3 Inflammasome in Carotid Atherosclerosis. J. Stroke Cerebrovasc. Dis..

[200] Xiao H., Lu M., Lin T.Y., Chen Z., Chen G., Wang W.-C., Marin T., Shentu T., Wen L., Gongol B. (2013). Sterol Regulatory Element Binding Protein 2 Activation of NLRP3 Inflammasome in Endothelium Mediates Hemodynamic-Induced Atherosclerosis Susceptibility. Circulation.

[201] Zheng F., Xing S., Gong Z., Xing Q. (2013). NLRP3 Inflammasomes Show High Expression in Aorta of Patients with Atherosclerosis. Heart Lung Circ..

[202] Jin Y., Fu J. (2019). Novel Insights into the NLRP3 Inflammasome in Atherosclerosis. J. Am. Heart Assoc..

[203] Chamberlain J., Evans D., King A., Dewberry R., Dower S., Crossman D., Francis S. (2006). Interleukin-1β and Signaling of Interleukin-1 in Vascular Wall and Circulating Cells Modulates the Extent of Neointima Formation in Mice. Am. J. Pathol..

[204] Menu P., Pellegrin M., Aubert J.-F., Bouzourene K., Tardivel A., Mazzolai L., Tschopp J. (2011). Atherosclerosis in ApoE-Deficient Mice Progresses Independently of the NLRP3 Inflammasome. Cell Death Dis..

[205] Kirii H., Niwa T., Yamada Y., Wada H., Saito K., Iwakura Y., Asano M., Moriwaki H., Seishima M. (2003). Lack of Interleukin-1β Decreases the Severity of Atherosclerosis in ApoE-Deficient Mice. Arterioscler. Thromb. Vasc. Biol..

[206] Parathath S., Mick S.L., Feig J.E., Joaquin V., Grauer L., Habiel D.M., Gassmann M., Gardner L.B., Fisher E.A. (2011). Hypoxia Is Present in Murine Atherosclerotic Plaques and Has Multiple Adverse Effects on Macrophage Lipid Metabolism. Circ. Res..

[207] Folco E.J., Sheikine Y., Rocha V.Z., Christen T., Shvartz E., Sukhova G.K., Di Carli M.F., Libby P. (2011). Hypoxia but Not Inflammation Augments Glucose Uptake in Human Macrophages: Implications for Imaging Atherosclerosis with 18Fluorine-Labeled 2-Deoxy-D-Glucose Positron Emission Tomography. J. Am. Coll. Cardiol..

[208] Folco E.J., Sukhova G.K., Quillard T., Libby P. (2014). Moderate Hypoxia Potentiates Interleukin-1β Production in Activated Human Macrophages. Circ. Res..

[209] Li X., Zhang Y., Xia M., Gulbins E., Boini K.M., Li P.-L. (2014). Activation of Nlrp3 Inflammasomes Enhances Macrophage Lipid-Deposition and Migration: Implication of a Novel Role of Inflammasome in Atherogenesis. PLoS ONE.

[210] Bergsbaken T., Fink S.L., Cookson B.T. (2009). Pyroptosis: Host Cell Death and Inflammation. Nat. Rev. Microbiol..

[211] Ridker P.M., Everett B.M., Thuren T., MacFadyen J.G., Chang W.H., Ballantyne C., Fonseca F., Nicolau J., Koenig W., Anker S.D. (2017). Antiinflammatory Therapy with Canakinumab for Atherosclerotic Disease. N. Engl. J. Med..

[212] Grebe A., Hoss F., Latz E. (2018). NLRP3 Inflammasome and the IL-1 Pathway in Atherosclerosis. Circ. Res..

[213] Ridker P.M., Rane M. (2021). Interleukin-6 Signaling and Anti-Interleukin-6 Therapeutics in Cardiovascular Disease. Circ. Res..

[214] Abderrazak A., Couchie D., Mahmood D.F.D., Elhage R., Vindis C., Laffargue M., Matéo V., Büchele B., Ayala M.R., El Gaafary M. (2015). Anti-Inflammatory and Antiatherogenic Effects of the NLRP3 Inflammasome Inhibitor Arglabin in ApoE2.Ki Mice Fed a High-Fat Diet. Circulation.

[215] Ma Y., Long Y., Chen Y. (2021). Roles of Inflammasome in Cigarette Smoke-Related Diseases and Physiopathological Disorders: Mechanisms and Therapeutic Opportunities. Front. Immunol..

[216] Mehta S., Dhawan V. (2020). Molecular Insights of Cigarette Smoke Condensate-Activated NLRP3 Inflammasome in THP-1 Cells in a Stage-Specific Atherogenesis. Int. Immunopharmacol..

[217] Yao Y., Mao J., Xu S., Zhao L., Long L., Chen L., Li D., Lu S. (2019). Rosmarinic Acid Inhibits Nicotine-Induced C-Reactive Protein Generation by Inhibiting NLRP3 Inflammasome Activation in Smooth Muscle Cells. J. Cell Physiol..

[218] Ozkok A. (2019). Cholesterol-Embolization Syndrome: Current Perspectives. Vasc. Health Risk Manag..

[219] Saric M., Kronzon I. (2011). Cholesterol Embolization Syndrome. Curr. Opin. Cardiol..

[220] Satish M., Agrawal D.K. (2020). Atherothrombosis and the NLRP3 Inflammasome—Endogenous Mechanisms of Inhibition. Transl. Res..

[221] Kato J., Tsuruda T., Kita T., Kitamura K., Eto T. (2005). Adrenomedullin. Arterioscler. Thromb. Vasc. Biol..

[222] Morgenthaler N.G., Struck J., Alonso C., Bergmann A. (2005). Measurement of Midregional Proadrenomedullin in Plasma with an Immunoluminometric Assay. Clin. Chem..

[223] Khan S.Q., O’Brien R.J., Struck J., Quinn P., Morgenthaler N., Squire I., Davies J., Bergmann A., Ng L.L. (2007). Prognostic Value of Midregional Pro-Adrenomedullin in Patients with Acute Myocardial Infarction: The LAMP (Leicester Acute Myocardial Infarction Peptide) Study. J. Am. Coll. Cardiol..

[224] Koyama T., Kuriyama N., Suzuki Y., Saito S., Tanaka R., Iwao M., Tanaka M., Maki T., Itoh H., Ihara M. (2021). Mid-Regional pro-Adrenomedullin Is a Novel Biomarker for Arterial Stiffness as the Criterion for Vascular Failure in a Cross-Sectional Study. Sci. Rep..

[225] Melander O., Newton-Cheh C., Almgren P., Hedblad B., Berglund G., Engström G., Persson M., Smith J.G., Magnusson M., Christensson A. (2009). Novel and Conventional Biomarkers for the Prediction of Incident Cardiovascular Events in the Community. JAMA.

[226] Roos M., Schuster T., Ndrepepa G., Baumann M., Lutz J., Braun S., Martinof S., Schömig A., Heemann U., Kastrati A. (2012). Association of Midregional Proadrenomedullin with Coronary Artery Stenoses, Soft Atherosclerotic Plaques and Coronary Artery Calcium. Heart Vessel..

[227] Yoshihara F., Ernst A., Morgenthaler N.G., Horio T., Nakamura S., Nakahama H., Nakata H., Bergmann A., Kangawa K., Kawano Y. (2007). Midregional Proadrenomedullin Reflects Cardiac Dysfunction in Haemodialysis Patients with Cardiovascular Disease. Nephrol. Dial. Transplant..

[228] Brouwers F.P., de Boer R.A., van der Harst P., Struck J., de Jong P.E., de Zeeuw D., Gans R.O., Gansevoort R.T., Hillege H.L., van Gilst W.H. (2012). Influence of Age on the Prognostic Value of Mid-Regional pro-Adrenomedullin in the General Population. Heart.

[229] Neumann J.T., Tzikas S., Funke-Kaiser A., Wilde S., Appelbaum S., Keller T., Ojeda-Echevarria F., Zeller T., Zwiener I., Sinning C.R. (2013). Association of MR-Proadrenomedullin with Cardiovascular Risk Factors and Subclinical Cardiovascular Disease. Atherosclerosis.

[230] Sawada S. (2021). Cystatin C as a Promising Biomarker of Atherosclerotic Plaque. J. Atheroscler. Thromb..

[231] Shi G.P., Sukhova G.K., Grubb A., Ducharme A., Rhode L.H., Lee R.T., Ridker P.M., Libby P., Chapman H.A. (1999). Cystatin C Deficiency in Human Atherosclerosis and Aortic Aneurysms. J. Clin. Investig..

[232] Nishimura Y., Honda K., Yuzaki M., Tajima K., Nakamura R., Nakanishi Y., Kaneko M., Agematsu K., Nagashima M. (2021). Serum Cystatin C Level as a Biomarker of Aortic Plaque in Patients with an Aortic Arch Aneurysm. J. Atheroscler. Thromb..

[233] Zhu Y., Zhang H.-P., Wang Y.-C., Ren T.-T., Li J., Xu M.-L., Wang X.-Q., Liu F.-C., Lau A., Wen Y.-F. (2015). Serum Cystatin C Level Is Associated with Carotid Intima-Media Thickening and Plaque. Scand. J. Clin. Lab. Investig..

[234] Correa S., Morrow D.A., Braunwald E., Davies R.Y., Goodrich E.L., Murphy S.A., Cannon C.P., O’Donoghue M.L. (2018). Cystatin C for Risk Stratification in Patients After an Acute Coronary Syndrome. JAHA.

[235] Colley K.J., Wolfert R.L., Cobble M.E. (2011). Lipoprotein Associated Phospholipase A2: Role in Atherosclerosis and Utility as a Biomarker for Cardiovascular Risk. EPMA J..

[236] Ballantyne C.M., Hoogeveen R.C., Bang H., Coresh J., Folsom A.R., Chambless L.E., Myerson M., Wu K.K., Sharrett A.R., Boerwinkle E. (2005). Lipoprotein-Associated Phospholipase A2, High-Sensitivity C-Reactive Protein, and Risk for Incident Ischemic Stroke in Middle-Aged Men and Women in the Atherosclerosis Risk in Communities (ARIC) Study. Arch. Intern. Med..

[237] Ballantyne C.M., Hoogeveen R.C., Bang H., Coresh J., Folsom A.R., Heiss G., Sharrett A.R. (2004). Lipoprotein-Associated Phospholipase A2, High-Sensitivity C-Reactive Protein, and Risk for Incident Coronary Heart Disease in Middle-Aged Men and Women in the Atherosclerosis Risk in Communities (ARIC) Study. Circulation.

[238] Cai A., Zheng D., Qiu R., Mai W., Zhou Y. (2013). Lipoprotein-Associated Phospholipase A2 (Lp-PLA_2): A Novel and Promising Biomarker for Cardiovascular Risks Assessment. Dis. Markers.

[239] Papapanagiotou A., Siasos G., Kassi E., Gargalionis A.N., Papavassiliou A.G. (2015). Novel Inflammatory Markers in Hyperlipidemia: Clinical Implications. Curr. Med. Chem..

[240] Bonnefont-Rousselot D. (2016). La Lp-PLA2, marqueur d’inflammation vasculaire et de vulnérabilité de la plaque d’athérosclérose. Ann. Pharm. Françaises.

[241] Hassan M. (2015). STABILITY and SOLID-TIMI 52: Lipoprotein Associated Phospholipase A2 (Lp-PLA2) as a Biomarker or Risk Factor for Cardiovascular Diseases. Glob. Cardiol. Sci. Pract..

[242] Maiolino G., Bisogni V., Rossitto G., Rossi G.P. (2015). Lipoprotein-Associated Phospholipase A2 Prognostic Role in Atherosclerotic Complications. World. J. Cardiol..

[243] Kolodgie F.D., Burke A.P., Skorija K.S., Ladich E., Kutys R., Makuria A.T., Virmani R. (2006). Lipoprotein-Associated Phospholipase A2 Protein Expression in the Natural Progression of Human Coronary Atherosclerosis. Arter. Thromb. Vasc. Biol..

[244] Mannheim D., Herrmann J., Versari D., Gössl M., Meyer F.B., McConnell J.P., Lerman L.O., Lerman A. (2008). Enhanced Expression of Lp-PLA2 and Lysophosphatidylcholine in Symptomatic Carotid Atherosclerotic Plaques. Stroke.

[245] Vickers K.C., Maguire C.T., Wolfert R., Burns A.R., Reardon M., Geis R., Holvoet P., Morrisett J.D. (2009). Relationship of Lipoprotein-Associated Phospholipase A2 and Oxidized Low Density Lipoprotein in Carotid Atherosclerosis. J. Lipid Res..

[246] Zhang H., Gao Y., Wu D., Zhang D. (2020). The Relationship of Lipoprotein-Associated Phospholipase A2 Activity with the Seriousness of Coronary Artery Disease. BMC Cardiovasc. Disord..

[247] Nagase H., Visse R., Murphy G. (2006). Structure and Function of Matrix Metalloproteinases and TIMPs. Cardiovasc. Res..

[248] Rodriguez J.A., Orbe J., Martinez de Lizarrondo S., Calvayrac O., Rodriguez C., Martinez-Gonzalez J., Paramo J.A. (2008). Metalloproteinases and Atherothrombosis: MMP-10 Mediates Vascular Remodeling Promoted by Inflammatory Stimuli. Front. Biosci..

[249] Myasoedova V.A., Chistiakov D.A., Grechko A.V., Orekhov A.N. (2018). Matrix Metalloproteinases in Pro-Atherosclerotic Arterial Remodeling. J. Mol. Cell. Cardiol..

[250] Olejarz W., Łacheta D., Kubiak-Tomaszewska G. (2020). Matrix Metalloproteinases as Biomarkers of Atherosclerotic Plaque Instability. Int. J. Mol. Sci..

[251] MacColl E., Khalil R.A. (2015). Matrix Metalloproteinases as Regulators of Vein Structure and Function: Implications in Chronic Venous Disease. J. Pharm. Exp..

[252] Purroy A., Roncal C., Orbe J., Meilhac O., Belzunce M., Zalba G., Villa-Bellosta R., Andrés V., Parks W.C., Páramo J.A. (2018). Matrix Metalloproteinase-10 Deficiency Delays Atherosclerosis Progression and Plaque Calcification. Atherosclerosis.

[253] Matilla L., Roncal C., Ibarrola J., Arrieta V., García-Peña A., Fernández-Celis A., Navarro A., Álvarez V., Gainza A., Orbe J. (2020). A Role for MMP-10 (Matrix Metalloproteinase-10) in Calcific Aortic Valve Stenosis. ATVB.

[254] Mohler E.R., Gannon F., Reynolds C., Zimmerman R., Keane M.G., Kaplan F.S. (2001). Bone Formation and Inflammation in Cardiac Valves. Circulation.

[255] Bossé Y., Miqdad A., Fournier D., Pépin A., Pibarot P., Mathieu P. (2009). Refining Molecular Pathways Leading to Calcific Aortic Valve Stenosis by Studying Gene Expression Profile of Normal and Calcified Stenotic Human Aortic Valves. Circ. Cardiovasc. Genet..

[256] Jung J.-J., Razavian M., Challa A.A., Nie L., Golestani R., Zhang J., Ye Y., Russell K.S., Robinson S.P., Heistad D.D. (2015). Multimodality and Molecular Imaging of Matrix Metalloproteinase Activation in Calcific Aortic Valve Disease. J. Nucl. Med..

[257] Yan Y., Zhang J.-W., Zang G.-Y., Pu J. (2019). The Primary Use of Artificial Intelligence in Cardiovascular Diseases: What Kind of Potential Role Does Artificial Intelligence Play in Future Medicine?. J. Geriatr. Cardiol..

[258] Turing A.M., Epstein R., Roberts G., Beber G. (2009). Computing Machinery and Intelligence. Parsing the Turing Test: Philosophical and Methodological Issues in the Quest for the Thinking Computer.

[259] Ghahramani Z. (2015). Probabilistic Machine Learning and Artificial Intelligence. Nature.

[260] Krittanawong C., Zhang H., Wang Z., Aydar M., Kitai T. (2017). Artificial Intelligence in Precision Cardiovascular Medicine. J. Am. Coll. Cardiol..

[261] Munger E., Hickey J.W., Dey A.K., Jafri M.S., Kinser J.M., Mehta N.N. (2021). Application of Machine Learning in Understanding Atherosclerosis: Emerging Insights. APL Bioeng..

[262] Huynh-Thu V.A., Saeys Y., Wehenkel L., Geurts P. (2012). Statistical Interpretation of Machine Learning-Based Feature Importance Scores for Biomarker Discovery. Bioinformatics.

[263] Jain P.K., Sharma N., Saba L., Paraskevas K.I., Kalra M.K., Johri A., Laird J.R., Nicolaides A.N., Suri J.S. (2021). Unseen Artificial Intelligence-Deep Learning Paradigm for Segmentation of Low Atherosclerotic Plaque in Carotid Ultrasound: A Multicenter Cardiovascular Study. Diagnostics.

[264] Hampe N., Wolterink J.M., van Velzen S.G.M., Leiner T., Išgum I. (2019). Machine Learning for Assessment of Coronary Artery Disease in Cardiac CT: A Survey. Front. Cardiovasc. Med..

[265] Boi A., Jamthikar A.D., Saba L., Gupta D., Sharma A., Loi B., Laird J.R., Khanna N.N., Suri J.S. (2018). A Survey on Coronary Atherosclerotic Plaque Tissue Characterization in Intravascular Optical Coherence Tomography. Curr. Atheroscler. Rep..

[266] Munger E., Choi H., Dey A.K., Elnabawi Y.A., Groenendyk J.W., Rodante J., Keel A., Aksentijevich M., Reddy A.S., Khalil N. (2020). Application of Machine Learning to Determine Top Predictors of Noncalcified Coronary Burden in Psoriasis: An Observational Cohort Study. J. Am. Acad. Derm..

[267] Forné C., Cambray S., Bermudez-Lopez M., Fernandez E., Bozic M., Valdivielso J.M. (2020). The NEFRONA investigators Machine Learning Analysis of Serum Biomarkers for Cardiovascular Risk Assessment in Chronic Kidney Disease. Clin. Kidney J..

[268] Ross E.G., Shah N.H., Dalman R.L., Nead K.T., Cooke J.P., Leeper N.J. (2016). The Use of Machine Learning for the Identification of Peripheral Artery Disease and Future Mortality Risk. J. Vasc. Surg..

[269] Flores A.M., Demsas F., Leeper N.J., Ross E.G. (2021). Leveraging Machine Learning and Artificial Intelligence to Improve Peripheral Artery Disease Detection, Treatment, and Outcomes. Circ. Res..

[270] Qian Y., Zhang L., Sun Z., Zang G., Li Y., Wang Z., Li L. (2021). Biomarkers of Blood from Patients with Atherosclerosis Based on Bioinformatics Analysis. Evol. Bioinform. Online.

[271] Correia M., Kagenaar E., van Schalkwijk D.B., Bourbon M., Gama-Carvalho M. (2021). Machine Learning Modelling of Blood Lipid Biomarkers in Familial Hypercholesterolaemia versus Polygenic/Environmental Dyslipidaemia. Sci. Rep..

